# A patient-centric modeling framework captures recovery from SARS-CoV-2 infection

**DOI:** 10.1038/s41590-022-01380-2

**Published:** 2023-01-30

**Authors:** Hélène Ruffieux, Aimee L. Hanson, Samantha Lodge, Nathan G. Lawler, Luke Whiley, Nicola Gray, Tui H. Nolan, Laura Bergamaschi, Federica Mescia, Lorinda Turner, Aloka de Sa, Victoria S. Pelly, The Cambridge Institute of Therapeutic Immunology and Infectious Disease-National Institute of Health Research (CITIID-NIHR) BioResource COVID-19 Collaboration, Prasanti Kotagiri, Nathalie Kingston, John R. Bradley, Elaine Holmes, Julien Wist, Jeremy K. Nicholson, Paul A. Lyons, Kenneth G. C. Smith, Sylvia Richardson, Glenn R. Bantug, Christoph Hess

**Affiliations:** 1 MRC Biostatistics Unit, University of Cambridge, Cambridge Biomedical Campus, Cambridge, UK; 2 Cambridge Institute of Therapeutic Immunology and Infectious Disease, Jeffrey Cheah Biomedical Centre, University of Cambridge, Cambridge, UK; 3 Department of Medicine, University of Cambridge, Addenbrooke’s Hospital, Cambridge, UK; 4 Australian National Phenome Centre, Health Futures Institute, Murdoch University, Perth, Western Australia Australia; 5 Center for Computational and Systems Medicine, Health Futures Institute, Murdoch University, Perth, Western Australia Australia; 6 Perron Institute for Neurological and Translational Science, Nedlands, Western Australia Australia; 7 Department of Haematology, University of Cambridge, Cambridge Biomedical Campus, Cambridge, UK; 8 NIHR BioResource, Cambridge University Hospitals NHS Foundation, Cambridge Biomedical Campus, Cambridge, UK; 9 Department of Metabolism, Digestion and Reproduction, Faculty of Medicine, Imperial College London, London, UK; 10 Chemistry Department, Universidad del Valle, Cali, Colombia; 11 Institute of Global Health Innovation, Imperial College London, London, UK; 12 Department of Biomedicine, University and University Hospital Basel, Basel, Switzerland; 13 Botnar Research Centre for Child Health (BRCCH) University Basel & ETH Zurich, Basel, Switzerland

**Keywords:** Infectious diseases, Infectious diseases

## Abstract

The biology driving individual patient responses to severe acute respiratory syndrome coronavirus 2 infection remains ill understood. Here, we developed a patient-centric framework leveraging detailed longitudinal phenotyping data and covering a year after disease onset, from 215 infected individuals with differing disease severities. Our analyses revealed distinct ‘systemic recovery’ profiles, with specific progression and resolution of the inflammatory, immune cell, metabolic and clinical responses. In particular, we found a strong inter-patient and intra-patient temporal covariation of innate immune cell numbers, kynurenine metabolites and lipid metabolites, which highlighted candidate immunologic and metabolic pathways influencing the restoration of homeostasis, the risk of death and that of long COVID. Based on these data, we identified a composite signature predictive of systemic recovery, using a joint model on cellular and molecular parameters measured soon after disease onset. New predictions can be generated using the online tool http://shiny.mrc-bsu.cam.ac.uk/apps/covid-19-systemic-recovery-prediction-app, designed to test our findings prospectively.

## Abstract (web summary)

Ruffieux, Hess and colleagues analyze longitudinal phenotyping of patients with coronavirus disease 2019 to show that covariation of innate immune cell numbers, kynurenine metabolites and lipid metabolites influence the restoration of homeostasis, the risk of death and that of long COVID.

## Main

Coronavirus disease 2019 (COVID-19), which is caused by the severe acute respiratory syndrome coronavirus 2 (SARS-CoV-2), has a wide spectrum of clinical manifestations and has led to over 6.6 million deaths worldwide by the end of 2022 (ref. ^1^). When acute infection is resolved, health is restored in most individuals, yet some develop prolonged symptoms, known as long COVID^2–4^. SARS-CoV-2 can induce an important acute-phase reaction (systemic inflammation), profound changes in organismal metabolism and alterations across many elements of the immune system^5,6^. Evolution of these parameters over time is highly heterogeneous between patients^7,8^. How failure to restore organismal homeostasis relates to recovery from acute infection and development of long COVID remains unclear.

In this study, we exploited existing and new data from a previously described COVID-19 cohort^9^. Published results using this cohort focused on the acute and convalescent phases of illness using clinical, immune cell and inflammation parameters measured up to 3 months after disease onset. Here we adopted a wider perspective to understand organismal recovery, based on the same parameters as before^9^ (immune cell subsets, serum cytokines and C-reactive protein (CRP) levels) but collected over an extended follow-up period of 12 months, as well as newly established data (polar metabolites, glycoproteins and lipoproteins), and patient questionnaires addressing long-term symptoms of disease. We devised a patient-centric longitudinal modeling framework, based on functional principal component analysis (FPCA), to examine the cellular, metabolic and inflammatory drivers of patient variability in disease trajectories, and study recovery from COVID-19 in a broad, systemic sense.

## Results

### Cellular and metabolic changes associate with inflammation

We recruited 215 SARS-CoV-2 PCR-positive patients (hereafter COVID-19 patient, CovP) between 31 March 2020 and 7 August 2020 in Cambridge Hospitals (Wuhan strain). CovPs were categorized according to five severity classes based on peak clinical severity: A, asymptomatic (*n* = 18); B, mild symptomatic (*n* = 40); C, hospitalized without supplemental oxygen (*n* = 50); D, hospitalized with supplemental oxygen (*n* = 38); and E, hospitalized with assisted ventilation (*n* = 69). Symptomatic CovPs had onset of disease between 20 February 2020 and 18 June 2020, and hospital outcome (discharged, other hospital or facility, or deceased) for inpatients occurred after a median of 23 d after symptom onset (interquartile range (IQR), 32.25 d). Briefly, inpatient classes (C to E) were sampled at enrollment, approximately weekly up to 4 weeks, and then every 2 weeks up to 12 weeks. Discharged CovPs were asked to provide a follow-up sample 4–8 weeks after enrollment. Outpatient classes (A and B) were sampled at enrollment and subsequently after approximately 2 and 4 weeks. Participant recall beyond the original study period^9^ occurred at approximately 3, 6 and 12 months following recruitment. Additionally, 14 CovPs were newly included, after discharge from hospital. CRP levels, five serum cytokines (interferon (IFN)-γ, interleukin (IL)-10, IL-1β, IL-6, tumor necrosis factor (TNF)), 36 polar metabolites, 103 glycoproteins and lipoproteins, and 33 immune cell subsets were quantified from the blood samples collected at the above time points. Follow-up questionnaires^10^ assessing long-term symptoms were obtained from symptomatic CovPs (classes B to E) between 3 and 11 months after symptom onset (up to three questionnaires per CovP, median interval between two consecutive questionnaires for a same CovP: 5 months, IQR 1.5 month). The cohort also comprised 45 uninfected healthcare workers (hereafter healthy control, HC), defined by negative PCR test and serology, for whom blood samples were obtained at enrollment only.

**Extended Data Fig. 1:**
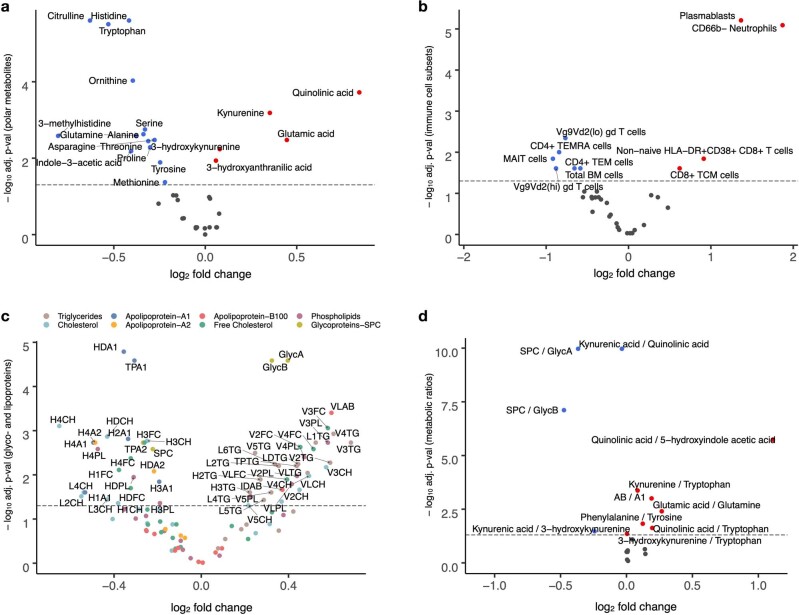
Volcano plots for differentially abundant biological parameters, comparing COVID- positive patients with HCs, using linear mixed modeling accounting for repeated subject observations (n = 215 CovPs, n = 45 HCs). **a**. Immune cell subsets. x-axis, log_2_ fold-change; y-axis, −log_10_ adjusted p-value; dashed horizontal line, 5% FDR threshold. Red and blue indicate significant up- resp. downregulation in CovPs vs HCs. **b**. Polar metabolites. Axes and colors as in a. **c**. Main classes of glyco- & lipoproteins. Axes as in a. Colors refer to compounds. **d**. Metabolic ratios. Axes and colors as in a.

**Extended Data Fig. 2:**
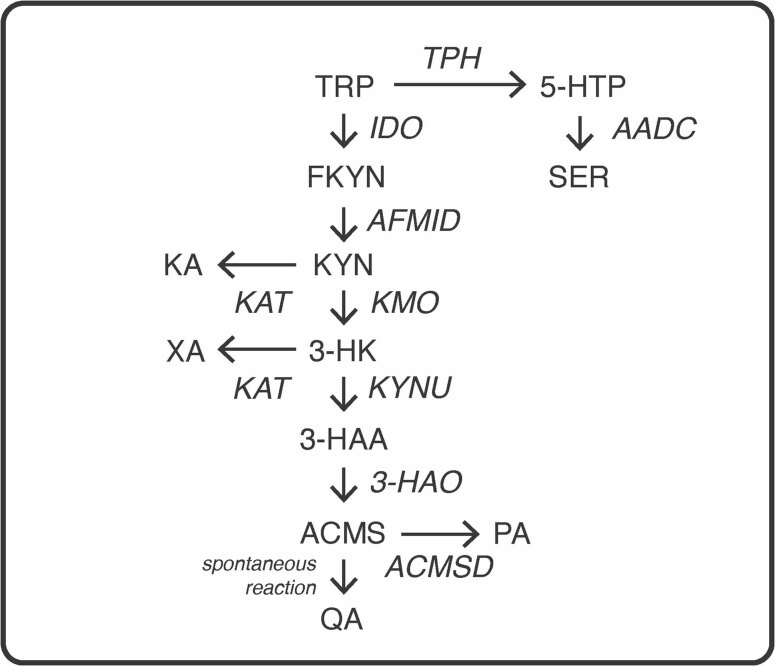
The kynurenine and serotonin pathways. *Metabolites*: TRP - tryptophan, FKYN - formyl-kynurenine, KYN - kynurenine, 3-HK - 3-hyroxykynurenine, 3-HAA - 3-hydroxyanthranillic acid, ACMS - 2-amino-3-carboxymuconate-6-semialdehyde, QA - quinolinic acid, KA - kynurenic acid, XA - xanthurenic acid, PA - picolinic acid, 5-HTP - 5-hydroxy-L-tryptophan, SER - serotonin. *Enzymes*: IDO - indoleamine-2,3-dioxygenase, AFMID - kynurenine aryl formidase, KMO - kynurenine-3-monooxygenase, KYNU - kynureninase, 3-HAO - 3-hydroxyanthranilate dioxy-genase, ACMSD - amino-b-carboxymuconate-semialdehyde-decarboxylase, KAT - kynurenine aminotransferase, TPH - tryptophan hydroxylase, AADC - aromatic-L-amino acid-L-tryptophan decarboxylase.

**Extended Data Fig. 3:**
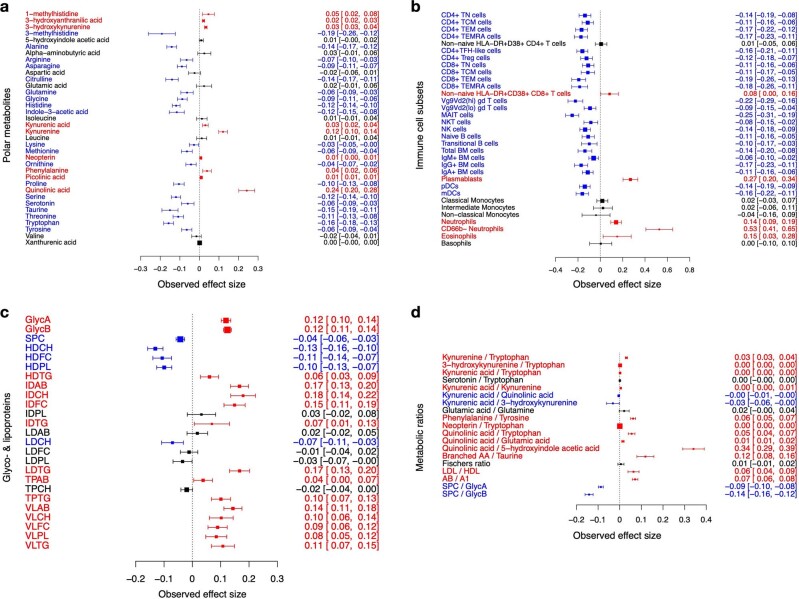
Association effects between biological parameters and CRP abundance with 95% confidence intervals (n = 215 CovPs, n = 45 HCs). **a**. Immune cell subsets. Red and blue indicate positive and negative associations, respectively, at FDR 5%. Effect sizes are not on the original scale as both the dependent (biological parameter) and independent (CRP) variables have been log-transformed. **b**. Polar metabolites. Colors and scale as in a. **c**. Main classes of glyco- & lipoproteins. Colors and scale as in a. **d**. Metabolic ratios. Colors and scale as in a.

First, to relate immunologic and metabolic changes in our cohort to findings from the published literature^5,6^, we examined the impact of SARS-CoV-2 infection across clinical, metabolic and cellular variables over a 7-week window after positive swab or symptom onset. Univariate linear mixed models were used, accounting for repeated participant measurements within the 7-week window, with each biologic parameter modeled as the dependent variable. A differential abundance analysis between HCs and CovPs across all severity groups indicated widespread systemic dysregulation (Extended Data Fig. 1). CovPs had a metabolic signature characterized by increased expression of intermediates from the kynurenine pathway (3-hydroxykynurenine, kynurenine, quinolinic acid) and depletion of the upstream amino acid tryptophan (Extended Data Figs. 1 and 2). Compared to HCs, plasma from CovPs also showed reduced abundance of other amino acids, most pronounced for citrulline, histidine and ornithine (Extended Data Fig. 1). There was further a decrease in high-density lipoproteins compared to HCs, in particular apolipoproteins, and an increase in very-low-density lipoproteins, in particular triglycerides and free cholesterol, and in the *N*-acetyl glycoprotein signals GlycA and GlycB (Extended Data Fig. 1). Absolute numbers of neutrophils, plasmablasts and activated CD8^+^ T cells were increased in CovPs compared to HCs, while absolute counts of Vγ9 Vδ2 γδ T cells, MAIT cells, CD4^+^ T_EMRA_ cells, CD4^+^ T_EM_ cells and total B_M_ cells (marginal zone-like B cells) were decreased (Extended Data Fig. 1), as previously reported^9^. Mixed-model association analyses, again accounting for serial participant measurements within the first 7 weeks after symptom onset or positive swab, revealed significant linear relationships between CRP concentration in serum—an indicator of the acute-phase response in COVID-19 patients^11,12^—and most metabolic and cellular variables in CovPs across all severity classes (A to E; Extended Data Fig. 3). Together, these analyses captured marked molecular and cellular abnormalities across the first 7 weeks after disease onset, and extensive covariation of these abnormalities with CRP.

### Functional principal component analysis captures heterogeneity of patient C-reactive protein trajectories

**Fig. 1:**
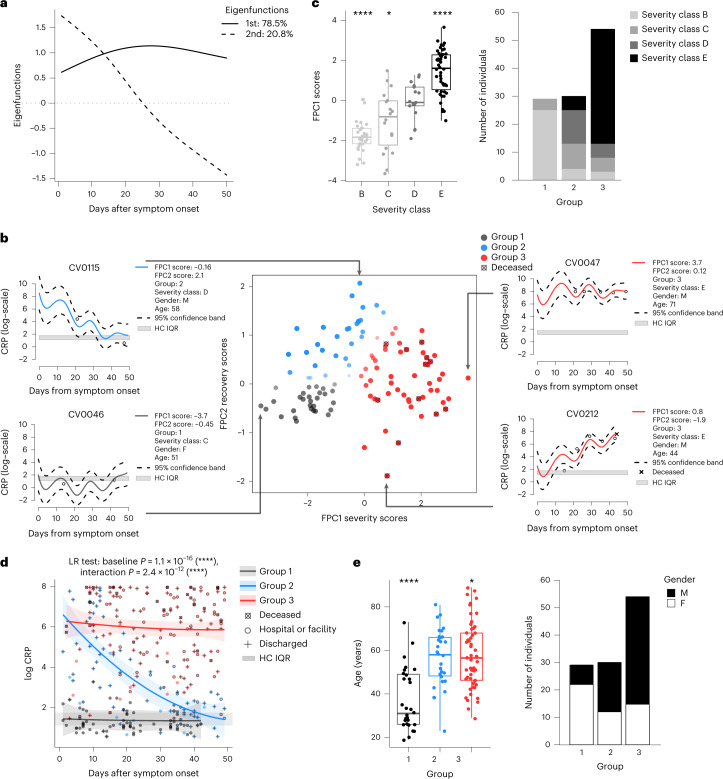
Functional principal component analysis of COVID-19 patients’ C-reactive protein levels. **a**, The first two eigenfunctions represent the severity of inflammation and the recovery from inflammation, respectively, over the 7-week window of the FPCA, accounting for 78.5% and 20.8% of the variability, respectively. **b**, Scatterplot showing the FPCA scores, FPC1 and FPC2, corresponding to the first and second eigenfunctions, respectively; each point corresponds to one CovP. *x* axis indicates FPC1 ‘severity’ scores (the higher the more severe the inflammation); *y* axis indicates FPC2 ‘recovery’ scores (the higher the more pronounced the temporal resolution of inflammation). ‘Recovery’ groups 1, 2 and 3 were obtained by Gaussian mixture modeling; the opacity and diameter of the points are proportional to the estimated probability of assignment of each CovP to their recovery group. Log-transformed CRP trajectories for four CovPs with extreme severity or recovery scores (left and right). Gray bands, normal CRP levels corresponding to the IQR of HCs’ (log-transformed) CRP levels. Points correspond to the observed values; red, blue and gray curves, trajectories estimated using the FPCA framework; dashed curves delineate the 95% confidence bands (*n* = 113 CovPs). **c**, FPCA scores and groups by CovP clinical severity classes (B, screening symptomatic; C, hospital no oxygen required; D, hospital supplemental oxygen; E, hospital assisted ventilation). In the box plots, the center line indicates the median, box limits represent the upper and lower quartiles and whiskers indicate 1.5 times the IQR. One-versus-all two-sided *t*-tests: ****P < 0.0001, ***P < 0.001, **P < 0.01 and *P < 0.05; overall: analysis of variance (ANOVA) *P* < 0.0001, *n* = 113 CovPs. **d**, CRP trajectories conditional on the recovery groups 1–3 with 95% confidence bands, estimated with a longitudinal mixed model accounting for patients’ repeated measurements (likelihood ratio (LR) tests for the baseline group effect and group × time interaction effect, significance labels as in **c**). Points correspond to observed values. Group 1, inflammation absent or mild; group 2, early, resolving inflammation; group 3, persisting inflammation. **e**, Characterization of the recovery groups by age (one-versus-all two-sided *t*-tests, ANOVA *P* < 0.0001) and gender (Fisher exact test *P* = 0.0001). Box plots and significance labels as in **c** (*n* = 113 CovPs).

Exploring study populations fails to provide patient-level insight. To overcome this limitation, we applied an FPCA framework to the parameter signatures discussed above. Based on the premise that inflammation is a central disruptor of homeostatic functions, we first aimed to identify the main modes of variation of CRP trajectories in CovPs over the first 7 weeks after symptom onset. Time points of analysis for asymptomatic CovPs (class A) were measured from the date of their first positive swab, while time points for symptomatic CovPs (classes B to E) were measured from the date of their symptom onset. As such, in order to prevent bias due to temporal shifts across participant trajectories (Supplementary Figs. 1 and 2), and ease the interpretation of the notion of ‘recovery’, we only analyzed the data from the 113 symptomatic CovPs whose CRP was measured at least twice. We used FPCA to model the CRP trajectory of each CovP as a mean function, reflecting the average CRP course across all CovPs, plus a truncated sum of patient-specific random deviations from the mean. These deviations were represented as a linear combination of orthonormal eigenfunctions, weighted by patient-specific scores, where the eigenfunctions and scores were estimated from the CovPs’ CRP data at all time points within 7 weeks after symptom onset. The first two eigenfunctions jointly accounted for more than 99% of the variance and, along with their corresponding scores, they were interpretable in terms of disease profiles: the first eigenfunction and associated scores (FPC1) acted as a proxy for inflammation severity (Fig. 1a,b). CovPs with positive FPC1 scores had higher CRP than the average CRP levels of all CovPs over the 7-week period (for example, CovP CV0047; Fig. 1b), while CovPs with negative FPC1 scores had lower CRP than the average CovP levels (for example, CovP CV0046; Fig. 1b). This interpretation was independently corroborated by the strong association of the scores with the B to E severity classes (Fig. 1c), establishing the clinical relevance of our FPCA inferences. We thus refer to the FPC1 scores as ‘severity scores’. The second eigenfunction and associated scores (FPC2) acted as a proxy for recovery from inflammation, with CovPs that had large positive FPC2 scores showing a marked improvement of the inflammatory status over time (for example, CovP CV0115; Fig. 1b). By contrast, inflammation resolved more slowly, or became more severe, compared to the mean function in CovPs with negative FPC2 scores (for example, CovP CV0212; Fig. 1b). Hence, we refer to the FPC2 scores as ‘recovery scores’. Of note, of the 113 CovPs, 40 CovPs from class C, D or E had either proven or suspected secondary infection—mostly hospital-acquired bacterial pneumonia (Supplementary Figs. 3 and 4). Additional analyses indicated an association between this status (infection proven, suspected or not suspected) and severity and recovery scores (Supplementary Fig. 3), in line with the expectation that a secondary infection would further increase inflammation levels. However, the FPCA estimates for CovPs with no suspected secondary infection were robust to the inclusion of CovPs with super-infection in the inference framework (Supplementary Fig. 5).

Gaussian mixture model (GMM) clustering of the severity and recovery scores uncovered three patient groups (Fig. 1b) with distinct inflammation trajectories. Group 1 had absent or mild inflammation over the 7-week window after symptom onset; group 2 had early, resolving inflammation, while group 3 had persisting inflammation (Fig. 1d), confirming that the patient-level FPCA estimates encompassed information on the magnitude and temporal profile of systemic inflammation as captured by CRP. Of note, all 12 CovPs who died belonged to group 3 (Fig. 1b). As expected^13^, additional analyses indicated that the groups were significantly associated with age and gender (Fig. 1e). Notably, groups 1–3 were not only entirely driven by disease severity, but also by the type of recovery profile (Fig. 1b). In particular, CovPs in group 2 had higher than average recovery scores, and each of the three groups comprised CovPs from multiple severity classes (Fig. 1c and Supplementary Table 1). We therefore refer to groups 1–3 as ‘recovery groups’.

The GMM clustering estimated probabilities of assignment of CovPs to the groups. Inspection of these probabilities indicated that some CovPs near the group boundaries were weakly allocated, that is, their assigned recovery group was uncertain (Fig. 1b). However, sensitivity analyses evaluating the impact of potential misallocation on all group-level findings indicated that estimates were barely changed by purposely reassigning all weakly allocated CovPs to another group (Supplementary Figs. 6–11). Collectively, these analyses showed that variability in CRP trajectories could be decomposed into separate ‘severity’ and ‘recovery’ latent contributions to the patient inflammation profiles, which enabled identifying three patient recovery groups with distinct inflammation dynamics.

### Dynamics across biologic systems are coordinated

**Fig. 2:**
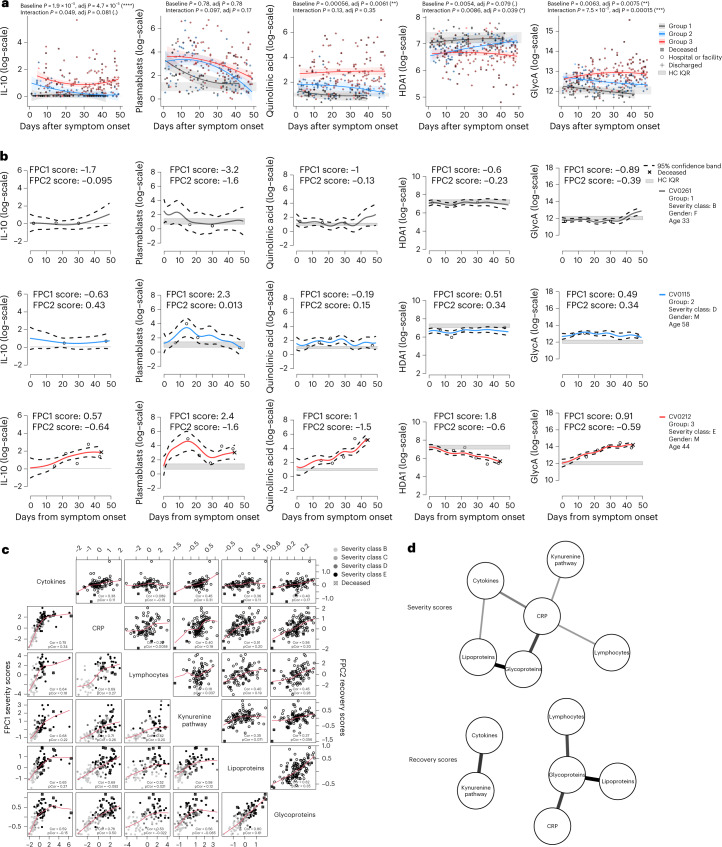
Group-level and patient-level estimates of cellular and molecular trajectories. **a**, Recovery-group trajectories estimated by longitudinal mixed modeling for five parameters, one from each data type (cytokines, lymphocytes, kynurenine-pathway metabolites, lipoproteins and glycoproteins) over the first 7 weeks after symptom onset, with 95% confidence bands. All levels have been log-transformed and the gray bands correspond to the IQR of HCs’ levels. False discovery rate (FDR)-adjusted *P* values from LR tests for baseline and interaction effects are indicated (****P < 0.0001, ***P < 0.001, **P < 0.01 and *P < 0.05). **b**, Trajectories of the same parameters as in **a**, estimated by FPCA for three CovPs, one from each recovery group. Dashed curves delineate the 95% confidence bands. **c**, Comparison of the severity (lower diagonal part) and recovery scores (upper diagonal part) obtained from the six FPCA studies with Pearson simple correlation (‘Cor’) and partial correlation (‘pCor’); significant estimates at an FDR level of 5% are highlighted in bold. Severity classes: B, screening symptomatic; C, hospital no oxygen required; D, hospital supplemental oxygen; E, hospital assisted ventilation. **d**, Conditional independence networks for the severity and recovery scores; an edge is shown if the pair of score types is directly associated (partial correlation) at an FDR level of 5%, and the opacity and width of the edge is proportional to the strength of the association.

**Extended Data Fig. 4:**
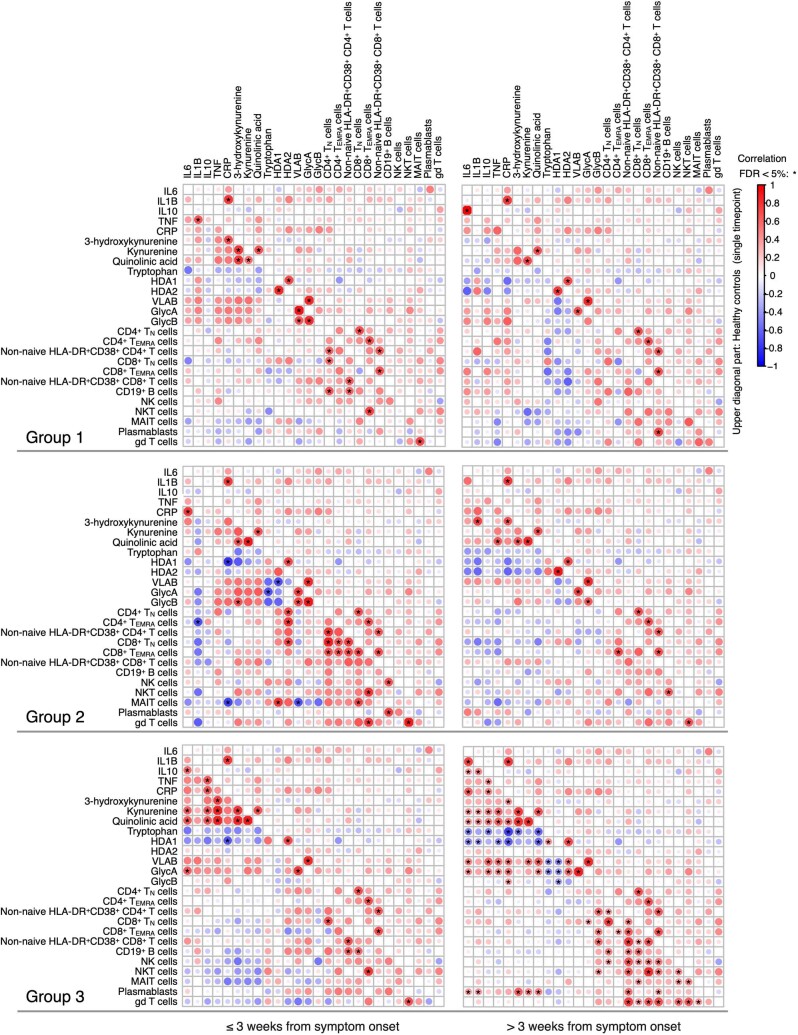
Correlation among the variables modeled by FPCA for the three recovery groups during acute phase (weeks 0-3) and convalescence (weeks 4–7). Lower diagonal part of all heatmaps, correlations in CovPs’ data. Upper diagonal part, correlations in HCs’ data (same for all heatmaps). Stars, significance of correlation tests at FDR 5%.

To test how longitudinal post-infection profiles, as reflected by recovery groups 1, 2 and 3, related to systemic recovery, we examined all biologic parameters quantified for the 113 symptomatic CovPs over the 7-week window following symptom onset. Mixed-effect modeling with time encoded as a continuous variable, indicated that the trajectories of the cellular and molecular parameters largely reflected the inflammation profile that characterized each recovery group, as exemplified by five parameters from each of the data types (Fig. 2a). However, correlation analyses conducted in each recovery group—stratifying the patient data into an acute-infection phase (weeks 0–3 after symptom onset) and a protracted-infection or convalescence phase (weeks 4–7 after symptom onset)—further indicated significant covariations across data types, for all recovery groups in both time bins, which were not observed in data from HCs (Extended Data Fig. 4). This suggested that, irrespective of disease severity, as a population, infected individuals did not make full immune and metabolic recovery by week 7 after symptom onset.

To explore the interplay between the temporal profiles of the inflammatory, immune cell and metabolomic responses at the patient level, we performed additional multivariate FPCA studies on sets of parameters whose alterations were identified to be signatures of active SARS-CoV-2 infection in our population-level analyses, that is, apolipoproteins (HDA1, HDA2, VLAB), glycoproteins (GlycA, GlycB), kynurenine-pathway metabolites (3-hydroxykynurenine, kynurenine, quinolinic acid, tryptophan), serum cytokines (IFN-γ, IL-10, IL-1β, IL-6, TNF) and subsets of lymphocytes (CD4^+^ T_EMRA_ cells, CD4^+^ T_N_ cells, non-naive HLA-DR^+^CD38^+^ CD4^+^ T cells, CD8^+^ T_EMRA_ cells, CD8^+^ T_N_ cells, non-naive HLA-DR^+^CD38^+^ CD8^+^ T cells, total B_M_ cells, γδ T cells, mucosal-associated invariant T (MAIT) cells, natural killer (NK) cells, NKT cells, plasmablasts). Trajectories reconstructed for three individual CovPs (CV0261, CV0115 and CV0212) from each recovery group largely co-evolved over the disease course (Fig. 2b). The temporal profiles for quinolinic acid in these CovPs broadly agreed with the group-level longitudinal estimates (Fig. 2a). More generally, the 95% confidence bands of the estimated trajectories for CovP CV0261 (group 1, symptomatic but not hospitalized) covered the normal levels (HCs’ IQR; Fig. 2b), suggesting absent or mild alterations. The trajectories of CovP CV0115 (group 2, supplemental oxygen) were slightly above or below normal levels, but with no sign of deterioration over time (Fig. 2b). Finally, CovP CV0212 (group 3, assisted ventilation; died 44 d after symptom onset) exhibited a clear deterioration in all parameters, with the estimated trajectories departing from normal levels (Fig. 2b). The trajectories of cellular-level, molecular-level and inflammation-level parameters, and the severity and recovery scores for all CovP analyzed in the FPCA framework, can be inspected interactively at http://shiny.mrc-bsu.cam.ac.uk/apps/covid-19-patient-trajectories/, along with basic anthropomorphic and clinical information on each CovP. These reconstructed trajectories indicated that, although individual parameter courses tended to be representative of the general parameter evolution within the groups, some CovPs displayed exceptional trajectories, emphasizing the value of patient-specific estimates to resolve the covariation of cellular and molecular parameters with inflammation at the patient level.

In addition, the FPCA studies on the cellular and metabolic parameters more broadly defined the interplay between data types in response to infection. Similarly to the CRP FPCA, the first and second eigenfunctions could be interpreted as proxies for ‘severity’ and ‘recovery’ (or ‘normalization’) of the parameter trajectories, respectively, and the different sets of severity scores were highly correlated across data types (Fig. 2c), confirming that inflammatory, immunologic and metabolomic alterations were closely interlinked. Inspecting the partial correlation structure among severity scores further indicated direct relationships between the CRP severity scores and all other severity scores, except for the lipoprotein scores (with which they were indirectly associated through the glycoprotein and cytokine scores; Fig. 2d), corroborating the central role of CRP scores in assessing severity profiles. The severity scores from all data types echoed the clinical severity classes (B to E; Fig. 2c), indicating that the variability in the various trajectories reflected pathophysiologic signatures relevant to clinical disease. Correlation of recovery scores across the different types of parameters was somewhat weaker, although still significant in most cases (Fig. 2c), which may reflect distinct dynamics between cellular compartments or differing half-lives. Alternatively, it could indicate persistent disruption of select biologic systems despite resolution of inflammation. Together, these data captured the complexity of the mostly coordinated dynamic changes observed across the immune, metabolic and inflammatory systems.

### Disruption of biologic parameters can last for months

**Fig. 3:**
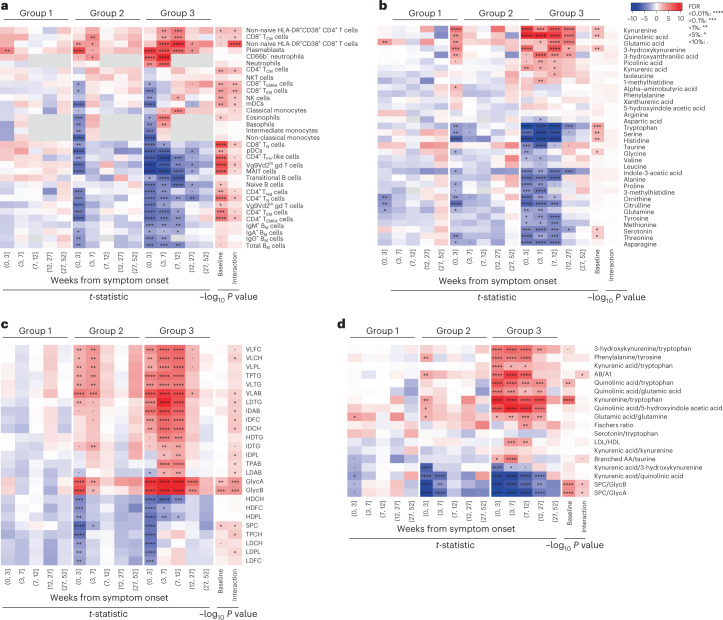
Long-term recovery-group trajectories for biologic parameters (*n* = 113 CovPs). **a**–**d**, Immune cell subsets (**a**), polar metabolites (**b**), glycoproteins and lipoproteins (**c**) and metabolic ratios (**d**) for each recovery group compared with the HC levels over a year after symptom onset, using mixed modeling. The first 5 × 3 columns indicate the *t*-statistics obtained for the group effect and corresponding significance after FDR multiplicity adjustment, and the last two columns indicate the significance (−log_10_ adjusted *P* value) of the baseline and interaction effects using the group-level longitudinal mixed models.

To examine the long-term dynamics of cellular and molecular recovery, we modeled differential parameter abundance between each recovery group and HCs over an extended time frame of 1 year from infection, using linear mixed models and binning the CovPs data into five time windows (weeks 0–3, weeks 4–7, weeks 8–12, weeks 13–27, weeks 28–52 after symptom onset; chosen so as to involve similar numbers of observations). As expected, group 1 (mild or absent inflammation) showed minor early disruptions, linked to plasmablasts, citrulline, glutamic acid, glutamine, ornithine and several metabolic ratios that were significantly different from HCs in weeks 0–3 after symptom onset (Fig. 3a–d). In group 2, many parameters (including several lipoproteins, glycoproteins and lymphocytes) were altered up to week 4 (67%) or week 8 (33%) after symptom onset, but were indistinguishable from HCs in the subsequent time windows (that is, from week 8 onwards; Fig. 3a–d), thus aligning with the recovery from inflammation defining the group. Group 3 (persistent inflammation) showed widespread and long-lasting cellular alterations. Most notably, plasmablast and non-naive HLA-DR^+^CD38^+^ CD8^+^T cell numbers were still increased, whereas the numbers of plasmacytoid dendritic cells, CD4^+^ follicular helper T-like cells, Vγ9 Vδ2^lo^ γδ T cells, MAIT cells, naive B cells and CD4^+^ regulatory T (T_reg_) cells were still decreased in the fourth time window (weeks 13–27; Fig. 3a). Similarly, there were persistent metabolic alterations (weeks 13–27) in group 3 compared to HCs, such as increased kynurenine, quinolinic acid and glutamic acid, and decreased levels of tryptophan, indole-3-acetic acid and serotonin (Fig. 3b), as well as increased levels of VLAB, GlycA and GlycB (Fig. 3c). Using group-level longitudinal mixed models with time modeled as a continuous variable, we next assessed baseline group effects (that is, group differences at symptom onset) and group × time interaction effects. Nearly half of the parameters included in this study had significant baseline and/or interaction effect(s) (for instance, NK cells, CD8^+^ T_EM_ cells, myeloid dendritic cells, CD8^+^ T_N_ cells, GlycA and GlycB displayed both baseline and interaction effects, CD4^+^ follicular helper T-like cells, Vγ9 Vδ2^lo^ γδ T cells, MAIT cells, kynurenine, quinolinic acid and tryptophan had significant baseline effects only, and HLA-DR^+^CD38^+^ CD8^+^ T cells had significant interaction effects only; Fig. 3a–c). These parameters, when measured early after infection, might therefore contain information regarding an individual’s ability to recover. In all, these results indicated that recovery groups 1–3 had dissimilar parameter recovery rates, with group-3 CovPs experiencing persisting biologic disruptions up to 6 months after symptom onset.

### Biologic recovery and clinical outcomes are interlinked

**Fig. 4:**
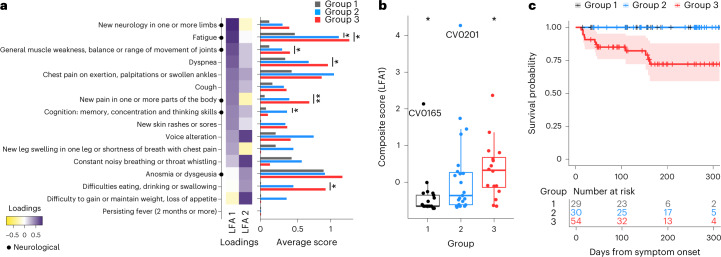
Long-term symptom characterization of the recovery groups by latent factor and survival analyses. **a**, Estimated loadings for the two latent factors (LFA1 and LFA2) underlying the long-COVID symptoms. The score for each symptom by recovery group is also shown (multiplicity-adjusted pairwise two-sided Wilcoxon tests). **b**, The first latent factor (LFA1) was used as a symptom composite score. Association with the recovery groups was assessed using one-versus-all two-sided Wilcoxon tests (****P < 0.0001, ***P < 0.001, **P < 0.01 and *P < 0.05) and overall Kruskal–Wallis test *P* = 0.0006. In the box plots, the center line is the median, box limits indicate the upper and lower quartiles and whiskers represent 1.5 times the IQR (*n* = 65 CovPs). **c**, Kaplan–Meier survival curves and table for the three recovery groups (*n* = 113 CovPs), with 95% confidence bands. The curves for groups 1 and 2 overlap (blue and black).

We next asked how biologic recovery profiles related to long COVID, using questionnaires on long-term symptoms (covering respiratory, neurological, gastrointestinal and other physical sequelae)^10^. These questionnaires were collected from 65 CovPs (54% female, median age 51 years old, IQR 29 years old) between months 2 and 11 after symptom onset. A comparison of the reported symptoms indicated that CovPs in group 3 reported more neurological symptoms (fatigue, muscle weakness, pain, difficulty eating, drinking, swallowing) compared to group 1 (Fig. 4a). Of note, in group 3, fatality and mechanical ventilation (a source of non-infection-related sequelae) added unavoidable limitations. We also conducted simple and partial correlation analyses (Supplementary Fig. 12), which indicated that the neurological symptoms were largely interconnected, with the exception of anosmia and dysgeusia. A latent factor analysis, which aimed to characterize the joint manifestation of the reported symptoms, identified two latent factors, the first of which (LFA1) appeared to be driven by the neurological symptoms. In particular, new neurology in limbs, fatigue and muscle weakness were attributed the largest loadings for LFA1 (Fig. 4a). As it accounted for most of the modeled variability, LFA1 also served as a natural patient-level latent proxy for long-term clinical manifestations, and was associated with the recovery groups (Fig. 4b). As such, LFA1 was significantly higher for CovPs from group 3 (poorer score), and lower for CovPs from group 1 (Fig. 4b). However, there was substantial variability within each recovery group. In particular, two patients, CV0165 in group 1 and CV0201 in group 2, had high composite scores, although their cellular, inflammatory and molecular trajectories had essentially returned to normal levels by week 7 after symptom onset, as estimated by the FPCA studies (and seen at http://shiny.mrc-bsu.cam.ac.uk/apps/covid-19-patient-trajectories/). This suggested that systemic, subjectively perceived sequelae persisted in these individuals despite absent, or rapidly resolving, inflammation and cellular/molecular disruption. Finally, a survival analysis found an increased risk of death for group 3 compared to groups 1 and 2 (log-rank test, *P* = 0.0007; Fig. 4c). Together, these results showed that the inflammatory response to infection, as encompassed by the three recovery groups, was tightly linked with survival outcomes and long-term clinical sequelae up to 1 year after symptom onset, despite substantial patient-to-patient variations.

### Joint model finds early markers of incomplete recovery

**Fig. 5:**
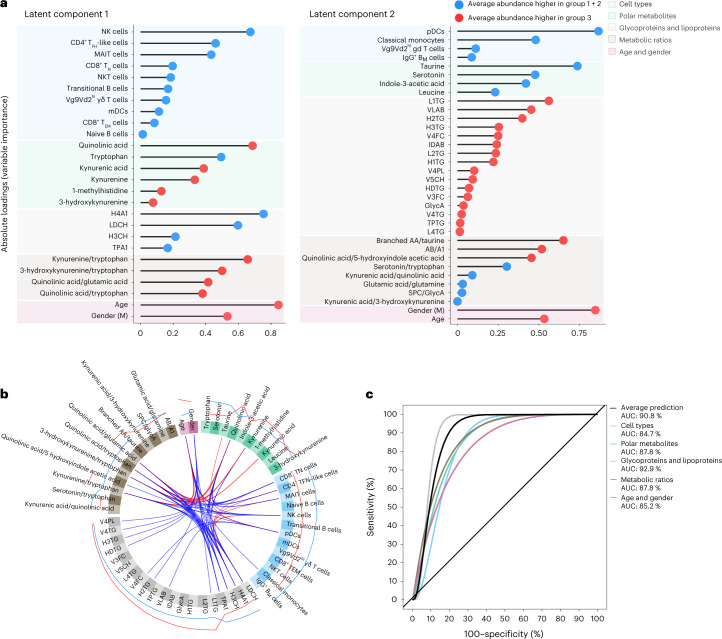
Predictive modeling for the recovery groups. **a**, Absolute loadings forming early predictive signatures, for the first and second latent components of the sGCCA, arranged per data type. The blue and red dots indicate an average abundance greater in groups 1 + 2 and group 3, respectively. **b**, Circular plot linking pairs of variables from the two signatures, if their absolute Pearson correlation exceeds 0.75. Red and blue indicating a positive and negative correlation, respectively. External blue and red lines show the relative average abundance of the selected variables within the two categories (blue indicates groups 1 + 2 and red indicates group 3). **c**, ROC curves for the predictive performance of sGCCA on the test set. The curves correspond to the average prediction based on the five data types (black), and the prediction based each data type separately (colors).

To test whether the type of recovery profile of individual CovPs could be predicted soon after infection, we trained an integrative model on samples collected during the early phase of the disease. Specifically, we applied a sparse generalized canonical correlation analysis (sGCCA) algorithm, extended for supervised analysis, on all cellular and molecular parameters jointly, also including age and gender as a separate canonical vector. sGCCA involved an internal selection procedure for identifying a subset of parameters predictive of incomplete recovery from the nearly 200 candidate markers. Briefly, sGCCA implemented a trade-off between maximizing the correlation of biologic parameters (immune cell subsets, polar metabolites, glycoproteins, lipoproteins and diverse metabolic ratios) and maximizing the discrimination between unfavorable recovery profiles (group 3) and favorable recovery profiles (group 1 and group 2 merged). We used the first sample from each CovP, provided it was taken within 3 weeks from symptom onset, and considered a training–test set split involving 70% and 30% of the samples, respectively. The signatures for the first two latent components (Fig. 5a) and the circular plot (Fig. 5b) were obtained using the training samples, while the receiver operating characteristic (ROC) curves (Fig. 5c) were obtained using the left-out test samples.

The sign of the average signature parameter abundances (Fig. 5a) aligned with published results^5,6,9^. NK cells appeared with the largest weight in the first signature (average cell numbers lower in CovPs with poor prognosis; Fig. 5a). Metabolic intermediates from the kynurenine pathway, and corresponding ratios, also appeared as important markers of the type of recovery (Fig. 5a), corroborating reports on the involvement of the pathway in adaptive immunity and inflammation^14^. Quinolinic acid, tryptophan, kynurenic acid, kynurenine and 3-hydroxykynurenine were selected in the first signature, while serotonin, a neurotransmitter derived from tryptophan catabolism, was selected in the second signature. These metabolites appeared together with a series of innate immune cells. The area under the curve (AUC) on the test set reached 90.8% (Fig. 5c). The predictive performance when restricting the signatures to each data type was also high (AUCs > 84.7%), suggesting strong interdependence of the biologic processes captured in our study. The AUC assessing the predictive value of age and gender was the second lowest (85.2%) after that of the cell-type block (84.7%), suggesting that the signal encompassed in the molecular markers has greater predictive value (>87.8%) compared to anthropomorphic characteristics only. Moreover, while age and gender already contain substantial information regarding the risk of incomplete recovery at the population level (Fig. 5c), at the individual level—that is, clinically most relevant—a low risk in terms of anthropomorphic factors does not rule out the possibility of incomplete organismal recovery, as our FPCA findings capture. For instance, the individual-level estimates for CovP CV0209, 38 years old, in group 3 (accessible at http://shiny.mrc-bsu.cam.ac.uk/apps/covid-19-patient-trajectories) indicated unfavorable molecular parameter trajectories and scores; this young CovP did not survive. Hence, relevant biologic markers measured early after disease onset in young individuals may correctly predict a poor outcome.

Finally, we developed an interactive tool to browse predictions based on selected markers from the two systemic recovery signatures available at http://shiny.mrc-bsu.cam.ac.uk/apps/covid-19-systemic-recovery-prediction-app/. For any new prediction run through this tool, an estimated prediction score conveys the level of confidence in the predicted recovery profile, and the predictive performance on the test set is re-evaluated based on the subset of markers supplied. This internal performance assessment of the sGCCA model on the Cambridge cohort suggested that the cellular and molecular markers from the estimated signatures contain valuable predictive information for anticipating incomplete recovery, already when measured soon after symptom onset.

## Discussion

Our joint modeling framework of the longitudinal immunologic, metabolic and clinical data on a SARS-CoV-2 cohort indicated protracted temporal covariation patterns that highlighted acute-phase inflammation as a common denominator interlinked with incomplete clinical, immunologic and metabolic recovery up to a year after disease onset, and suggested that coordinated dynamics of the innate immune system, kynurenine and host lipid metabolism were likely underpinning restoration of overall homeostasis. Specifically, the reconstruction of parameter trajectories at the patient level, and the estimation of patient-specific latent ‘severity’ and ‘recovery’ scores, allowed the characterization of the inter-patient and intra-patient variability in responses to infection, and revealed three types of patient profiles in an unsupervised fashion, agnostic of any clinical assessments subject to interpretation. These profiles constituted distinct patient ‘systemic recovery’ categories, which characterized disease course, risk of death and of long COVID beyond peak clinical severity. Furthermore, our patient-centric FPCA framework permitted confronting the biology of group profiles with that of individual patients (http://shiny.mrc-bsu.cam.ac.uk/apps/covid-19-patient-trajectories/). Finally, an early prognosis of recovery for a new patient can be obtained from our pilot predictive model (http://shiny.mrc-bsu.cam.ac.uk/apps/covid-19-systemic-recovery-prediction-app/), whose excellent performance in our cohort warrants independent validation to evaluate clinical actionability.

Our data suggested that a limited number of pathophysiologic processes impact many of the parameters appearing together in early composite signatures predictive of poor prognosis. Of note, both the inflammatory immunopathology of severe acute SARS-CoV-2 infection (note that upper airway viral persistence was excluded for all severity classes^9^), and hemophagocytic lymphohistiocytosis^15^, a cytokine storm syndrome, are characterized by elevated triglycerides—likely linked to protracted hyper-inflammation. NK cells play a central role in antiviral immunity through the secretion of pro-inflammatory cytokines and cytotoxic activity, and NK cell dysfunction is also a key criterion of hemophagocytic lymphohistiocytosis^15,16^. NK cells had the largest weight in the first predictive signature, with low absolute counts associated with poor outcome. This reduction of NK cells in peripheral blood from individuals with unresolving CRP (group 3) suggests an inflammation-driven perturbation of the NK cell compartment. The precise contribution of NK cell (dys)function to SARS-CoV-2-related acute and post-acute pathology remains unresolved^17–19^.

Pro-inflammatory cytokines activate the kynurenine pathway through induction of indoleamine 2,3-dioxygenase (IDO-1), as observed in our cohort^20^. Kynurenine-pathway activation has been implied in linking inflammation and central nervous system alterations by favoring the degradation of tryptophan toward 3‐hydroxykynurenine and quinolinic acid^21^, both of which appeared in the first predictive signature, and by reducing serotonin production^22^, which appeared in the second signature. It is plausible that abnormal levels of kynurenine-pathway intermediates, coupled with the significant reduction of serotonin abundance, contribute to neurological sequelae (for example, fatigue, weakness, chronic pain) of long COVID. Pathologic smell and taste, by contrast, may be related to more direct nerve sheath damage^23^. In addition, kynurenine and some of its metabolites are endogenous ligands of the transcription factor AhR. AhR is a regulator of balance between T_reg_ and the T_H_17 subset of helper T cells^24,25^, and binding of kynurenine to AhR in CD4^+^ T cells promotes the expression of the transcription factor FoxP3 and T_reg_ cell differentiation^26^. In addition, 3-hydroanthranillic acid—a kynurenine-pathway metabolite—also enhances T_reg_ cell differentiation^27^. It will be interesting to define how function, frequency and absolute T_reg_ cell counts integrate to impact the pathophysiology of COVID-19.

The Cambridge cohort involved unvaccinated patients infected by the Wuhan variant and therefore constituted a clean floor for studying the immunologic and metabolic response triggered by the original strain of the SARS-CoV-2 virus. However, our study does not reflect the current epidemiological situation, which is also a limitation. Importantly however, our statistical framework is transferable to new cohorts (vaccinated, treated), facilitating systematic comparative work. By extension, long COVID and Epstein–Barr virus (EBV)-associated post-acute-infection syndrome (EBV-PAIS) share symptoms, and EBV reactivation after SARS-CoV-2 infection has been described^28,29^. Assessing how EBV-PAIS and SARS-CoV-2-related EBV reactivation relate to disruption of immune and metabolic homeostasis, and interlinked disease processes, using our methodology may be of value.

Altogether, our framework allows studying the molecular and clinical correlates of organismal biologic recovery. It further offers patient-centric lenses to dissect the heterogeneity of the immune and metabolic responses to SARS-CoV-2 infection, which facilitates the formulation of mechanistic hypotheses as well as the development and testing of personalized intervention strategies.

## Methods

### Cohort and samples

The cohort and initial sampling timeline have been described in earlier work^9^. Late-time-point samples have subsequently been collected and new metabolic data have been quantified. Briefly, study participants were CovPs attending Addenbrooke’s Hospital, Royal Papworth Hospital NHS Foundation Trust or Cambridge and Peterborough Foundation Trust with a confirmed diagnosis of SARS-CoV-2 infection, as well as SARS-CoV-2-positive healthcare workers recruited from a staff screening program. The initial sampling program collected blood samples for 201 CovPs at enrollment (first samples collected on 31 March 2020), and at regular intervals up to 3 months after symptom onset, that is, inpatients were sampled approximately weekly up to 4 weeks after enrollment, then every 2 weeks up to 12 weeks after enrollment; discharged CovPs were asked to provide a follow-up sample 4–8 weeks after enrollment; outpatients were sampled after approximately 2 and 4 weeks after enrollment. Late-time-point samples were obtained up to a year after symptom onset, approximately at months 3, 6 and 12 following recruitment. A total of 14 additional hospitalized CovPs were included alongside the original cohort for the long-term follow-up after their discharge from Addenbrooke’s Hospital, providing late-time-point samples from month 3 after symptom onset onwards. Each participant was assigned to one of following categories of clinical severity: A, asymptomatic healthy workers (*n* = 18); B, symptomatic healthy workers (still working or self-isolating, *n* = 40); C, CovPs who presented to hospital but never required oxygen supplementation (*n* = 50); D, CovPs who were admitted to hospital and whose maximal respiratory support was supplemental oxygen (*n* = 38); E, CovPs who at some point required assisted ventilation (*n* = 69). Controls (*n* = 45) were SARS-CoV-2 PCR-negative hospital staff members with a negative serology. No statistical methods were used to predetermine sample sizes but our sample sizes are similar to those reported in previous publications^5,6^. No randomization or blinding was applicable as this was not an intervention study. Blood samples were drawn in EDTA, sodium citrate, serum and PAXgene Blood RNA tubes (BD Biosciences) and processed by the CITIID-NIHR COVID BioResource Collaboration group. No unique material was used in this study. All study participants provided written informed consent before enrollment. Ethics approval was obtained from the East of England–Cambridge Central Research Ethics Committee (‘NIHR BioResource’ REC ref. 17/EE/0025, and ‘Genetic variation AND Altered Leucocyte Function in health and disease–GANDALF’ REC ref. 08/H0308/176).

### C-reactive protein and cytokines

High-sensitivity CRP and serum cytokines (IFN-γ, IL-6, IL-10, IL-1β, TNF) were quantified by laboratories in Cambridge using standard assays^9^.

### Flow immunophenotyping and CyTOF assays

The assays are detailed in previous work on the early follow-up of the study cohort^9^. Briefly, peripheral blood mononuclear cells were obtained from peripheral venous blood collected in 10% sodium citrate tubes (up to 27 ml per sample). They were isolated using Leucosep tubes (Greiner Bio-One) with Histopaque 1077 (Sigma) by centrifugation at 800*g* for 15 min at room temperature. Peripheral blood mononuclear cells at the interface were collected, rinsed twice with autoMACS running buffer (Miltenyi Biotech) and cryopreserved in FBS with 10% dimethylsulfoxide. All samples were processed within 4 h of collection.

### Nuclear magnetic resonance spectroscopy and mass spectrometry based quantitative metabolic phenotyping

^1^H nuclear magnetic resonance (NMR) sample preparation was performed according to Bruker IVDr protocols^30^ and recommended procedures for IVDr metabolic analysis of COVID-19 plasma samples^31^. Plasma samples were stored at −80 °C until required, defrosted and centrifuged at 13,000*g* for 10 min at 4 ^°^C. The plasma supernatant was mixed with buffer (75 mM Na_2_HPO_4_, 2 mM NaN_3_, 4.6 mM sodium trimethylsilyl propionate-[2,2,3,3-2H_4_] in 80% deuterium oxide, pH 7.4 ± 0.1; 1:1 ratio), and transferred into a Bruker SampleJet NMR tube (5 mm). NMR measurements were performed on a Bruker 600 MHz Avance III HD spectrometer (IVDr) equipped with a BBI probe and fitted with Bruker SampleJet robot with the cooling system set to 5 °C. A quantitative calibration was completed before the analysis^32^. For each sample, a ^1^H one-dimensional experiment with solvent pre-saturation (32 scans, 98,304 data points, spectral width of 18,028.85 Hz) and a DIRE experiment was run (64 scans, 98,304 data points, spectral width of 18,028.85 Hz)^33^. Lipoprotein reports itemizing 112 lipoprotein parameters for each plasma sample were generated using the Bruker IVDr Lipoprotein Subclass Analysis (B.I.LISA) method^30^. ERETIC correction^34^ was applied to all DIRE spectra and spectra were calibrated by setting the spectral reference value to 0 (SR = 0). Integration of the alpha-1-acid glycoprotein *N*-acetyl signals GlycA and GlycB, and supramolecular phospholipid composite was performed using in-house scripts. GlycA, GlycB and supramolecular phospholipid composite integrations were performed using the following regions: *δ* 2.05–2.09, *δ* 2.09–2.12 and *δ* 3.15–3.35.

For biogenic amines, amino acids and tryptophan metabolic pathway analysis, plasma samples were thawed at 4 °C and prepared following methods previously reported^35,36^. For the quantification of biogenic amines and amino acid metabolites, separation was performed by ultra-high-performance liquid chromatography (UHPLC) using an Acquity UPLC (Waters) coupled to a Bruker impact II QToF mass analyzer (Bruker Daltonics). Resulting data files were processed for peak integration and quantification using Target Analysis for Screening Quantification (TASQ) software v2.2 (Bruker Daltonics). For the measurement of tryptophan and associated catabolites, separation was performed using an Acquity UPLC (Waters) coupled to a Xevo TQ-XS mass spectrometer (Waters). Raw files were processed for peak integrations and metabolite quantification using the TargetLynx package within MassLynx v4.2 (Waters). For both analytical assays, calibration curves were linearly fitted using a weighting factor of 1/*x* and quality control checks were performed.

### Data preprocessing and quality control

All statistical analyses were conducted using the R software^37^. Except for ratios for which no systematic transformation was applied, CRP, cytokines and all other cellular and molecular variables were log-transformed, using $x \mapsto {{{\mathrm{log}}}}_2\left( {x + 1} \right)$ (with the offset ‘+ 1’ accounting for zero counts while ensuring positivity), to stabilize variances and improve approximations of normality in statistical tests (although the normality assumption was not formally tested for each variable). For each molecular dataset, the presence of extreme measurements and/or batch effects was assessed using PCA visualization. No batch effect was observed. A standard box plot rule was applied to discard extreme samples, that is, with >20% of their measurements falling outside the Tukey outer fences. Following this procedure, two immune cell-type samples (1.1%) and ten metabolomic samples (1.6%) were removed from all downstream analyses (all glycoprotein and lipoprotein samples were retained).

### Differential abundance analysis and association with C-reactive protein

Differential abundance analysis between CovPs and HCs was conducted using linear mixed modeling to account for serial patient measurements over a window of 7 weeks after symptom onset or positive swab. Analyses also included gender and age as fixed effects. The following model was implemented using lmerTest R package (R notation):

$${{{\mathrm{dep}}}}\_{{{\mathrm{var}}}}\sim {{{\mathrm{covid}}}}\_{{{\mathrm{status}}}} + {{{\mathrm{age}}}} + {{{\mathrm{gender}}}} + (1|{{{\mathrm{subject}}}}\_{{{\mathrm{id}}}})$$

where the different molecular variables were taken in turn as the dependent variable dep_var and covid_status is a binary variable coding for ‘COVID positive’ or ‘COVID negative’. Significance of the covid_status effect was assessed using a type 3 *F* test and Satterthwaite’s method (to estimate the degrees of freedom for fixed effects), and adjustment for multiple testing across each molecular dataset was performed using an FDR level of 5%. When significance was reached, the molecular variable was called ‘upregulated’ or ‘downregulated’ based on the sign of the fold change.

The same linear mixed-model framework was used to test the association between CRP and each cellular/molecular variable, replacing the covid_status categorical variable with the quantitative (log-transformed) CRP variable.

Unless specified otherwise, all the analyses described hereafter are adjusted for multiple testing per data type, using an FDR level of 5%.

### Functional principal component analysis

FPCA was conducted to characterize the inter-patient and intra-patient variability and estimate individual disease trajectories using parameters reflecting inflammation, as well as the immunometabolic response to infection. These parameters were CRP levels, five cytokines (IFN-γ, IL-10, IL-1β, IL-6 and TNF), twelve lymphocyte subsets (CD4^+^ T_EMRA_ cells, CD4^+^ T_N_ cells, non-naive HLA-DR^+^CD38^+^ CD4^+^ T cells, CD8^+^ T_EMRA_ cells, CD8^+^ T_N_ cells, non-naive HLA-DR^+^CD38^+^ CD8^+^ T cells, total B_M_ cells, γδ T cells, MAIT cells, NK cells, NKT cells and plasmablasts), three lipoproteins (HDA1, HDA2 and VLAB), two glycoprotein signals (GlycA and GlycB) and four metabolites from the kynurenine pathway (3-hydroxykynurenine, kynurenine, quinolinic acid and tryptophan).

The R packages face^38^ and mfaces^39^ were used to implement the univariate FPCA study (for CRP levels) and multivariate FPCA studies (for all other groups of parameters listed above). Briefly, FPCA modeled the individual parameter trajectories as a mean function plus a truncated sum of random deviations from the mean, expressed as a linear combination of orthonormal eigenfunctions, weighted by patient-specific scores. In the multivariate setting, each parameter had a corresponding set of eigenfunctions but the scores were common to all parameters. Smooth estimates of variance and autocorrelation functions were also estimated as part of the FPCA framework, as well as cross-correlation functions in the multivariate setting. FPCA trajectories were estimated over a time window of 7 weeks after symptom onset. Asymptomatic individuals (severity class A) were not considered in the FPCA framework and all downstream analyses to avoid ambiguity when discussing recovery and to limit the risk of bias due to their different temporal alignment compared to individuals from the other classes (time was measured from the date of symptom onset for classes B to E, and from the date of first positive swab for class A; Supplementary Figs. 1 and 2).

### Gaussian mixture model clustering and sensitivity analysis for recovery-group assignment

GMM clustering with unconstrained covariances was performed on the CRP scores corresponding to the first two FPCA eigenfunctions to uncover groups of CovPs with similar disease trajectories. For the *n* individuals, the GMM estimated an *n* × *K* matrix of probabilities, whose (*i*, *k*)^th^ entry was the probability that subject *i* belonged to cluster *k*. The optimal classification was obtained by assigning each individual to the cluster corresponding to their highest probability.

To assess the impact of weakly assigned individuals on the subsequent group-level analyses, we conducted cluster membership sensitivity analyses by purposely misassigning individuals to clusters as follows: we identified all weakly assigned individuals to be individuals with maximum cluster probability below 0.8 (that is, for which the probability that they do not belong to their optimal recovery group exceeds 20%) and reassigned them to the cluster corresponding to the second-highest probability (that is, their ‘next best’ cluster). We then re-performed all analyses based on the resulting ‘perturbed’ recovery groups, and compared all estimates with those based on the original optimal recovery-group clustering (Supplementary Figs. 6–11).

Fisher’s exact tests were used to characterize the groups based on gender, and ANOVA and one-versus-all *t*-test (two-sided) were used to assess differences in age. For all subsequent analyses, significance labels are based on FDR-adjusted *P* values.

### Simple and partial correlation estimates

Correlation between the parameters considered in the FPCA studies (lymphocytes, lipoproteins, glycoproteins, kynurenine-pathway metabolites, cytokines and CRP levels) was assessed during acute infection (weeks 0–3 after symptom onset) and convalescence (weeks 4–7 after symptom onset; Extended Data Fig. 4). For each time window and each individual, the multiple measurements per person were averaged, before computing correlation within recovery groups. For HCs, samples were available at a single time point for each individual so a single correlation matrix was computed. FDR-adjusted correlation tests were performed using the R package TestCor separately for each recovery group and for the HCs.

Pairwise Pearson simple and partial correlation among the severity and recovery FPCA scores for lymphocytes, lipoproteins, glycoproteins, metabolites from the kynurenine pathway, as well as cytokines and CRP was also estimated.

### Longitudinal modeling

Univariate mixed models were used to estimate the temporal profile of each cellular/molecular parameter for the recovery groups 1, 2 and 3, that is, with random effects to account for serial measurements of patients. Polynomial splines of degree 2 were used to model the parameters with respect to the interaction between the time from symptom onset (time) and the recovery groups (group). The following model was fitted for each molecular variable (dep_var):

$${{{\mathrm{dep}}}}\_{{{\mathrm{var}}}}\sim {{{\mathrm{time}}}} \times {{{\mathrm{group}}}} + ({{{\mathrm{1}}}}|{{{\mathrm{subject}}}}\_{{{\mathrm{id}}}})$$

The significance of baseline effects (that is, difference between groups at time zero) and interaction effects (that is, difference in the group temporal courses) were tested using LR tests and adjusted for multiplicity across all variables of same data type.

Direct comparisons with HC levels were also conducted using mixed models whereby samples were grouped into five time windows (involving similar numbers of samples): (0, 3), (4, 7), (8, 12), (13, 27) and (28, 52) weeks after symptom onset. For each molecular variable and each time window, the following model was fitted:

$${{{\mathrm{dep}}}}\_{{{\mathrm{var}}}}\sim {{{\mathrm{category}}}} + ({{{\mathrm{1}}}}|{{{\mathrm{subject}}}}\_{{{\mathrm{id}}}})$$

where category is a categorical variable coding for the HC and three recovery groups, with the former used a reference factor level. To assess the discrepancy between each group’s parameters and HC parameters, the significance of the category levels was examined, adjusting for multiplicity over all parameters of same data type. Significance was not reported if ≤25 samples were available in the group and the time window under consideration.

### Long-term symptom analyses

Long-term symptoms were collected under the form of questionnaires given to CovPs between 2 and 11 months after symptom onset (average of 6 months). The questionnaires have been developed from Turner-Stokes et al.^10^, and were ethically reviewed for research use. The list of symptoms was: dyspnea; cough; chest pain on exertion; palpitations or swollen ankles; persisting fever (2 months or more); new leg swelling in one leg or shortness of breath with chest pain; new skin rashes or sores; voice alteration; difficulties eating, drinking or swallowing; constant noisy breathing or throat whistling; anosmia or dysgeusia; difficulty to gain or maintain weight, loss of appetite; new neurology in one or more limbs; new pain in one or more parts of the body; muscle weakness, balance or range of movement of joints; fatigue; cognition—memory, concentration and thinking skills. The questionnaire grades were recoded so the scores range from 0 (no symptom) to 5 (extreme manifestation of the symptom); the description of these grades for each symptom is detailed in the Supplementary Data Table 1. Up to three questionnaires per patient were collected, and the scores of CovPs with more than one questionnaire were averaged before the analysis. Correlation and partial correlation analyses were conducted on all symptoms apart from persisting fever (not experienced by any patient and hence a constant variable), adjusting for multiplicity (FDR 5%; Supplementary Fig. 12). Exploratory latent factor analysis by principal axes with oblique factor rotation was performed to characterize the joint manifestation of the symptoms, treating the data on the Likert scale. Nonparametric tests were used for overall comparison across recovery groups (Kruskal–Wallis rank-sum test), as well as for pairwise tests (two-sided Wilcoxon rank-sum tests).

### Survival analysis

The risk of death was studied by Kaplan–Meier survival analysis, using the three recovery groups 1, 2 and 3 as strata. Difference in survival between the groups was assessed using a log-rank test.

### Predictive modeling

Predictive modeling of systemic recovery was carried out to classify CovPs, shortly after disease onset, in terms of unfavorable (group 3) or favorable disease progression (groups 1 and 2, merged). An integrative sGCCA approach, adapted for supervised analysis, DIABLO^40^, was applied on the first sample of each patient from the cohort, provided that this sample was taken within 3 weeks after symptom onset. CovPs were randomly assigned to a training set or a left-out test set, according to a 70%–30% split (using the R package caret to balance the recovery-category distributions within the training and test sets). The sGCCA framework jointly accommodated all parameters across the different cellular and molecular data types (33 immune cell types, 36 polar metabolites, 103 glycoproteins and lipoproteins, and 19 selected metabolic ratios), as well as age and gender in a separate canonical vector. The training procedure used threefold cross-validation to select the numbers of predictors within the sparse latent components (composite predictive signatures).

### Systemic recovery-prediction tool

The predictive model based on CovPs’ early samples allows generating predictions for new samples collected when a patient presents to the clinic. An interactive tool to browse recovery prognoses is provided at http://shiny.mrc-bsu.cam.ac.uk/apps/covid-19-systemic-recovery-prediction-app/. For a new patient, each marker from the two signatures identified by the model (Fig. 5a) can be set in terms of percentiles of the empirical distribution formed by all Cambridge cohort patient and HC measurements. The colors appearing on the bar suggest normal ranges (gray, corresponding to the HCs’ IQR), low values (blue, smaller than the HCs’ first quartile) or high values (red, larger than the HCs’ third quartile). The initial values correspond to the median of HCs’ measurements. The tool runs the prediction based on the input values and it outputs the systemic recovery prognosis (1 + 2 or 3), along with a predicted score ranging from 0.5 to 1 and conveying the degree of confidence about the prediction (the larger, the higher the confidence).

As only a subset of markers from the two signatures may be quantified from blood tests collected in the clinic, the prediction can be based on a selection of markers chosen from the drop-down menus; the deselected markers are omitted in the linear combination corresponding to their latent component. To assess the expected performance of the model when restricted to a subset of markers, ROC curves are recomputed based on this subset using the Cambridge left-out test set (the curves are updated when selecting or deselecting markers). A poor performance on the test set (for example, AUC < 0.7) suggests that the selection of markers is insufficient to provide reliable predictions for the new patient. In that case, the predicted category should be disregarded and values for additional markers should be supplied where possible. As Vg9Vd2^hi^ γδ T appears in both signatures, its slider bar is displayed in the first column only.

### Reporting summary

Further information on research design is available in the Nature Portfolio Reporting Summary linked to this article.

## Online content

Any methods, additional references, Nature Portfolio reporting summaries, source data, extended data, supplementary information, acknowledgements, peer review information; details of author contributions and competing interests; and statements of data and code availability are available at 10.1038/s41590-022-01380-2.

## Supplementary information

Supplementary Information Supplementray Data 1–4 and Figures 1–14. Contains sensitivity analyses and complementary results: FPCA estimates for asymptomatic individuals; impact of secondary infections on the recovery profiles; recovery-group assignment sensitivity analysis; complement on the long-term symptom analyses.

Reporting Summary

Supplementary Table 1 Cohort characteristics by recovery groups 1–3. ‘Length of hospital stay’ is the number of days from hospital admission to discharge, transfer or death in hospital. sd, standard deviation.

Supplementary Data Table 1 Description of the grades for the long-term symptoms.

Supplementary Note

## Data Availability

The immune cell type, cytokine and CRP data, and the metadata for the early (0–3 months) follow-up^9^ are available at https://www.covid19cellatlas.org/patient/citiid/. The polar metabolite, glycoprotein and lipoprotein data for the full year post disease onset are available at 10.5281/zenodo.7277164.

## Code availability

The source code for the two R Shiny Apps can be accessed at 10.5281/zenodo.7527518 and 10.5281/zenodo.7528488. A repository with the scripts used for all analyses is also publicly accessible at 10.5281/zenodo.7527330.
