# Supplementary material for: A patient-centric modeling framework captures recovery from SARS-CoV-2 infection
Source: Nat Immunol. 2023 Jan 30;24(2):349–58. doi: 10.1038/s41590-022-01380-2 (PMC9892000; doi:10.1038/s41590-022-01380-2)
Supplement: Supplementary file 1 — Supplementray Data 1–4 and Figures 1–14. Contains sensitivity analyses and complementary results: FPCA estimates for asymptomatic individuals; impact of secondary infections on the recovery profiles; recovery-group assignment sensitivity analysis; complement on the long-term symptom analyses. [file 41590_2022_1380_MOESM1_ESM.pdf]

nature immunology

Resource

https://doi.org/10.1038/s41590-022-01380-2

A patient-centric modeling framework captures recovery from SARS-CoV-2 infection

In the format provided by the authors and unedited

Supplementary Data 1. FPCA estimates for asymptomatic subjects

For symptomatic patients (hereafter COVID-19 patient, CovP) from severity classes B to E, timepoints represented time from symptom onset. This definition could however not be directly applied to class-A CovPs, since they were asymptomatic when recruited; for them, timepoints were therefore defined based on the time from the first positive swab. These different definitions implied that we could not reliably align the disease trajectories of asymptomatic CovPs with those of symptomatic CovPs. While population-level analyses treating CovP observations as exchangeable repeated measures are unlikely to be sensitive to this, it is not the case of longitudinal analyses (treating time as a model parameter). For this reason, we excluded all class-A CovPs from the FPCA and subsequent analyses.

To provide more insight on the sensitivity of the FPCA framework to the inclusion of class-A CovPs, we re-conducted the analysis on all CovPs (i.e., including class A) and compared it to the original analysis, presented in the main text. Supplementary Fig. 1a,b indicates a strong agreement between the scores estimated by the two analyses, suggesting that the estimates for symptomatic CovPs would be barely impacted by the inclusion of the asymptomatic CovPs in the framework (two-sided correlation tests, severity scores: cor = 0.97, $p < 0.0001$; recovery scores: cor = 0.94, $p < 0.0001$). Supplementary Fig. 2a further indicates that FPC scores for class A largely overlap the scores from group-1 CovPs (i.e., with absent or mild inflammation), with 10 out of 15 class-A CovPs being predicted as group-1 CovPs by the Gaussian mixture model. However, the CRP profiles of the remaining class-A CovPs (2 predicted in group 2 and 3 in group 3) are appreciably different from healthy controls (hereafter HCs) (Supplementary Fig. 2b), the cause of which is difficult to determine given the absence of symptomatology and the lack of temporal alignment with the CovPs from the other classes. Of note, no secondary infection has been reported for these CovPs.

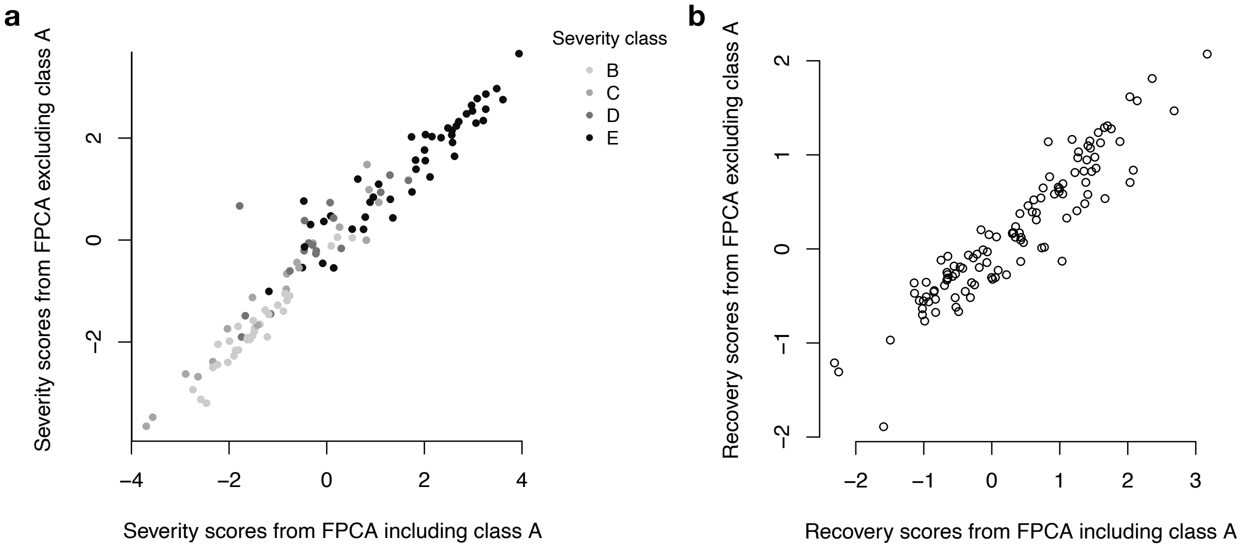

Supplementary Figure 1: **Sensitivity of the FPCA framework to the inclusion of asymptomatic CovPs. a.** Severity scores (FPC1) estimated from the data with (x-axis) and without (y-axis) asymptomatic CovPs from severity class A (the scores are shown for the subset of CovPs common to both analyses). **b.** As in a, but for the recovery scores (FPC2).

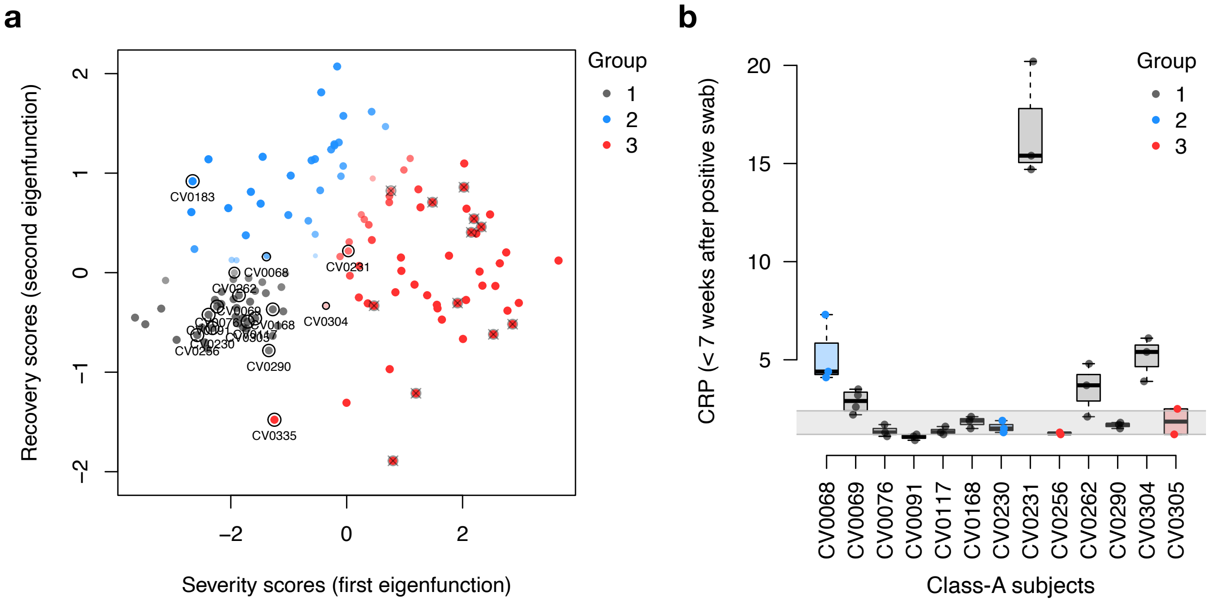

Supplementary Figure 2: **GMM prediction of recovery groups for asymptomatic CovPs. a.** Estimated FPC scores for asymptomatic CovPs (severity class A, black circles) overlaid on original FPC scores, along with their recovery groups (colors), as predicted by the Gaussian mixture model (GMM). **b.** Boxplots of the CRP measurements collected for the asymptomatic CovPs over the first 7 weeks after their first positive swab. Two CovPs (CV0183 from predicted group 2 and CV0335 from predicted group 3) are not shown as only two samples were collected for them within the 7-week window. Center line, median; box limits, upper and lower quartiles; whiskers, 1.5× IQR (n = 13 class-A CovPs). The colors correspond to the GMM-predicted recovery group and the grey horizontal band corresponds to the IQR of HCs' levels.

Supplementary Data 2. Impact of secondary infections on the recovery profiles

Among the CovPs analyzed in the FPCA framework, 13 had proven secondary infection and 27 had suspected secondary infection. Many infections were bacterial pneumonia, likely hospital-acquired for CovPs with assisted ventilation (severity class E) or with supplemental oxygen (severity class D); see Table 1.

The scores of CovPs with secondary infection were scattered among those of CovPs with no suspected infection (Supplementary Fig. 3a). The secondary infection status was, however, associated with the CRP FPC1 severity scores (anova: $p = 0.0071$, Supplementary Fig. 3b), in line with the expectation of inflammation levels being further increased by the additional insult. It was also associated with the recovery scores (anova: $p < 0.0001$, Supplementary Fig. 3b), whereby CovPs with suspected secondary infection had significantly higher scores, suggesting marked recovery trajectories. This may be explained as follows: (i) many CovPs with no secondary infection were from group 1 and had low recovery scores (as their inflammation trajectories were already within normal ranges), and (ii) nearly 70% of the CovPs with proven secondary infection were from group 3, with poorer recovery profiles (low recovery scores) that may be (partly?) imputable to the additional infection. Moreover, no CovP from recovery group 1 had proven or suspected infection and this pattern was significant (Fisher exact test: $p < 0.0001$, Supplementary Fig. 3c). However, secondary infections were not associated with poorer survival (Supplementary Fig. 3d).

Supplementary Fig. 4 shows examples of estimated trajectories conditioning on the secondary infection status. The marked recovery from inflammation of CovPs with suspected infection can

be clearly visualized from the CRP trajectory fits; recovery seems less pronounced for the other parameters shown.

Finally, to assess the impact of including CovPs with secondary infection in the FPCA framework, we re-applied the analyses to the subset of CovPs with no proven secondary infection. The estimated scores and those of our original analyses were in strong agreement, indicating that our framework is robust to the secondary infection status (Supplementary Fig. 5a,b).

	not suspected	suspected	proven
B	32	0	0
C	11	4	3
D	3	12	2
E	27	11	8

Table 1: **Secondary infection status by severity class.**

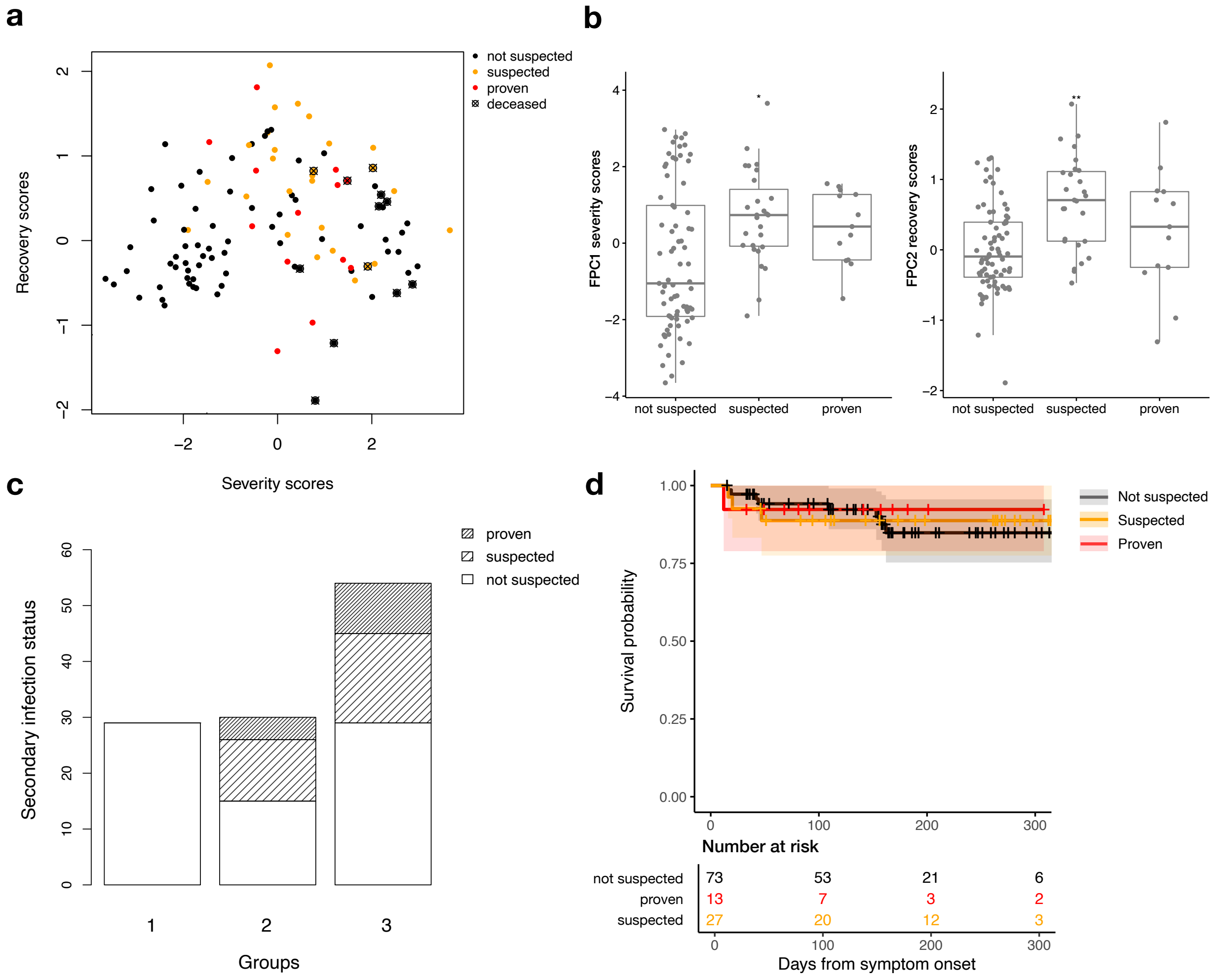

Supplementary Figure 3: **Secondary infection status and recovery groups. a.** CRP FPC scores, highlighting CovPs with suspected or proven secondary infection. **b.** Severity and recovery scores by secondary infection status, with one vs. all t-tests (stars) and overall anova tests $p = 0.007$ and $p < 0.0001$ for severity and recovery scores, respectively. Center line, median; box limits, upper and lower quartiles; whiskers, 1.5× IQR (n = 113 CovPs). **c.** Secondary infection status by recovery groups, with Fisher exact test. **d.** Survival probabilities Kaplan–Meier survival curves by secondary infection status, with log-rank test p-value.

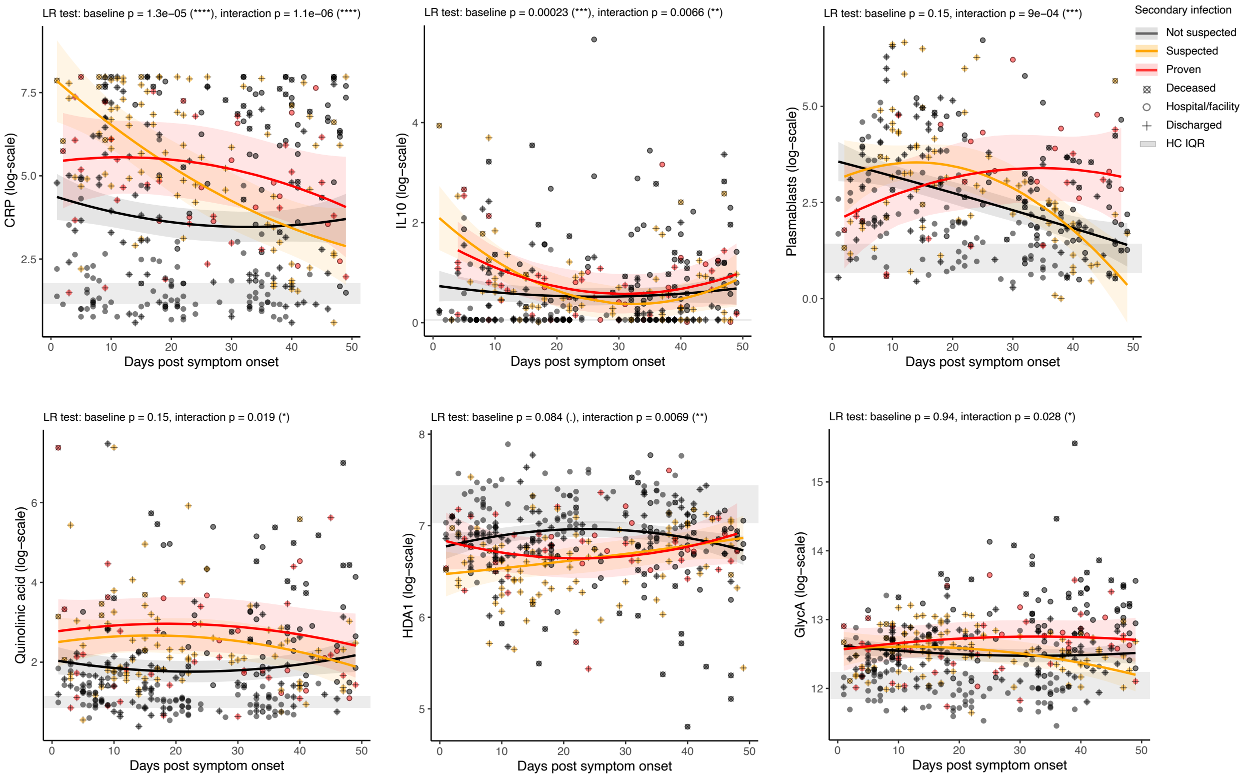

Supplementary Figure 4: **Trajectories by secondary infection status, estimated by longitudinal mixed modelling.** The plots show the fits for CRP and for one parameter per data type, with 95% confidence bands (same examples as in Fig. 2a of the main text). All levels have been log-transformed and the grey bands correspond to the IQR of HCs' levels; p-values from likelihood ratio tests for baseline and interaction effects are indicated.

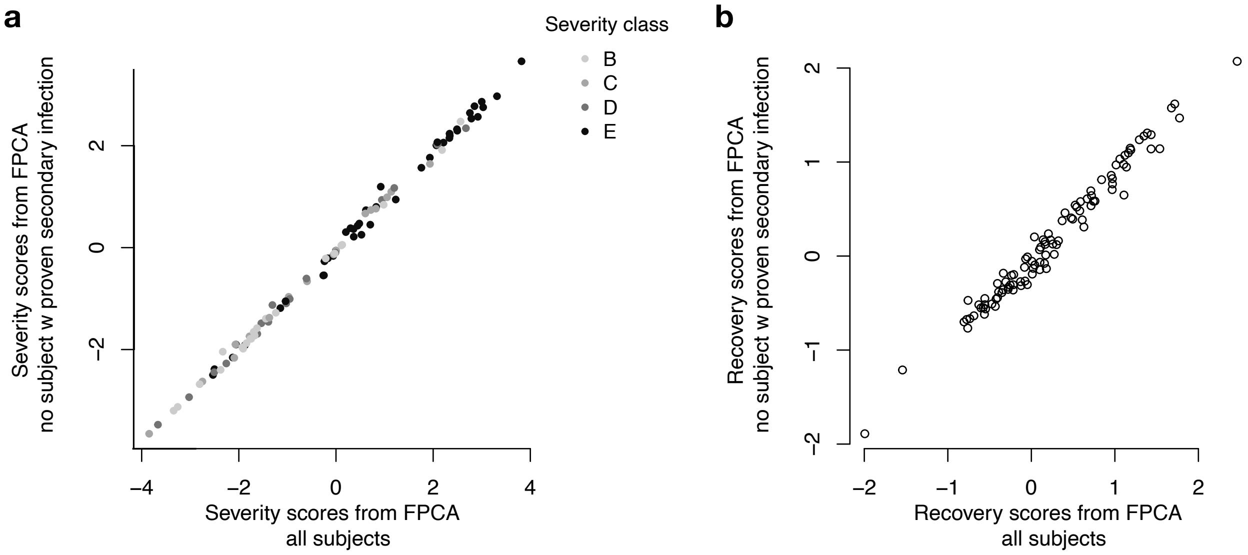

Supplementary Figure 5: **Sensitivity of the FPCA to the secondary infection status. a.** Scatterplot comparing the severity scores estimated from the data with (x-axis) or without (y-axis) CovPs with proven secondary infection (the scores are shown for the subset of CovPs common to both analyses). **b.** As in a, but for recovery scores.

Supplementary Data 3. Recovery group assignment sensitivity analysis

To assess the robustness our findings to the misassignment of CovPs to the recovery groups, we conducted a sensitivity study, re-performing all group-level analyses based on modified recovery groups, i.e., with suboptimal CovP assignment. Specifically, leveraging the soft boundaries of the Gaussian mixture model (each CovP belongs to the three groups with different degrees of belief), we identified the CovPs whose recovery-group membership probability was lower than 0.8 *(weakly assigned CovPs)* and re-assigned them to their "next best" recovery group, namely, to the cluster corresponding to their second highest probability. This led to a total of 17 CovPs changing groups (black circles at the group boundaries in Supplementary Fig. 6a,b, Table 2); note that no CovP moved from the mildest group 1 to the most severe group 3, or vice versa.

We then compared all subsequent group-level analyses based on these modified recovery groups to those based on the original, optimal recovery groups (i.e., as per the main text), namely:

- the group-level longitudinal fits: Supplementary Fig. 6c,d shows the example of log-transformed CRP trajectories; the two fits are visually very similar, and the baseline and interaction effects are highly significant in both cases. Similar observations hold when comparing the fits for the other cellular and molecular parameters;
- the group-level long-term recovery profiles (Fig. 3 of the main text): Figs 7, 8, 9 & 10 comparing the estimates for all windows and recovery groups again indicate a strong agreement, with discrepancies in reporting significance for just a few markers in specific time windows and groups;
- the association with long-term symptoms: the modified groups remained positively associated with the first latent factor (composite score, Kruskal–Wallis test, $p = 0.0001$);
- the survival analysis: one deceased CovP moved from group 3 to group 2 under the perturbed cluster assignment, however Supplementary Fig. 11a indicates that the poorer prognosis of group 3 remained significant;
- the prediction model: a few markers selected in signatures 1 and 2 in the original model were replaced by others in the model based on the modified recovery groups, i.e., for signature 1: Naive B cells $\leftrightarrow$ pDCs and H3CH $\leftrightarrow$ V5TG; for signature 2: Vg9Vd2(hi) $\gamma\delta$ T cells $\leftrightarrow$ NK cells and V5CH, V3FC, GlycA, TPTG $\leftrightarrow$ LDTG, V2FC, H4TG, H1A1. However these substitutions concerned markers with small absolute loadings, and Supplementary Fig. 11b indicates that the loadings of the remaining markers (shared by both models) were barely affected by the misassignment. Finally, the prediction performance on the left-out test set based on the modified recovery groups remained excellent (AUC $>$ 88.8 for all data types).

Modified recovery groups	1	2	3
Original recovery groups			
1	27	2	0
2	3	23	4
3	0	8	46

Table 2: **Assignment of CovPs to the recovery groups.** Original optimal CovPs grouping (rows) and grouping with misassignment of CovPs (columns).

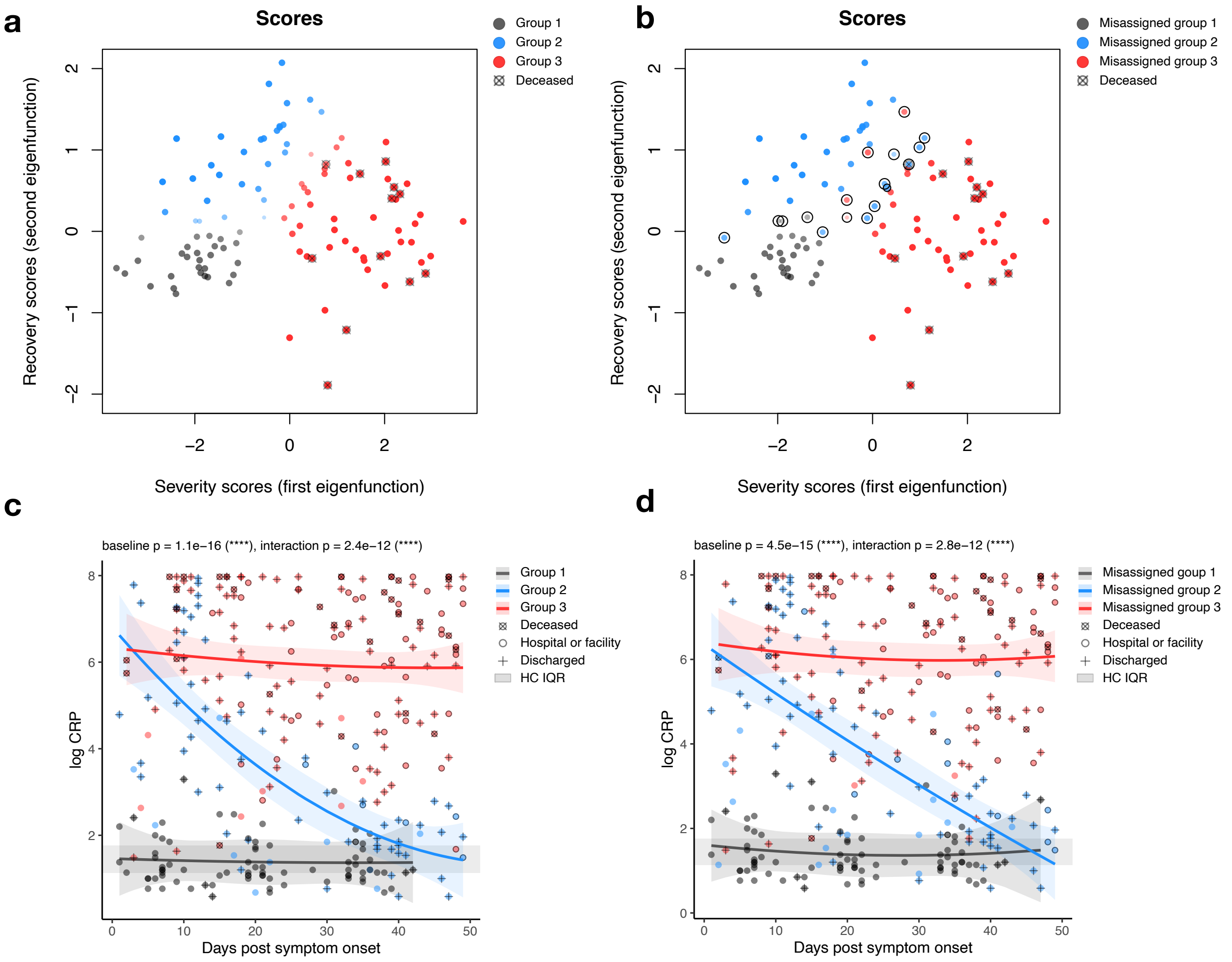

Supplementary Figure 6: **Sensitivity analysis for assignment of CovPs to recovery groups. a.** Clustering of FPC scores for the optimal grouping (same as Fig. 1b of the main text). **b.** Clustering of FPC scores for the modified grouping (recovery groups with misassigned CovPs). **c.** Group-level longitudinal analysis for the optimal grouping (same as Fig. 1d of the main text). Group baseline and group by time interaction effects indicated, along with significance level from likelihood ratio tests. **d.** Baseline and interaction effects as in c.

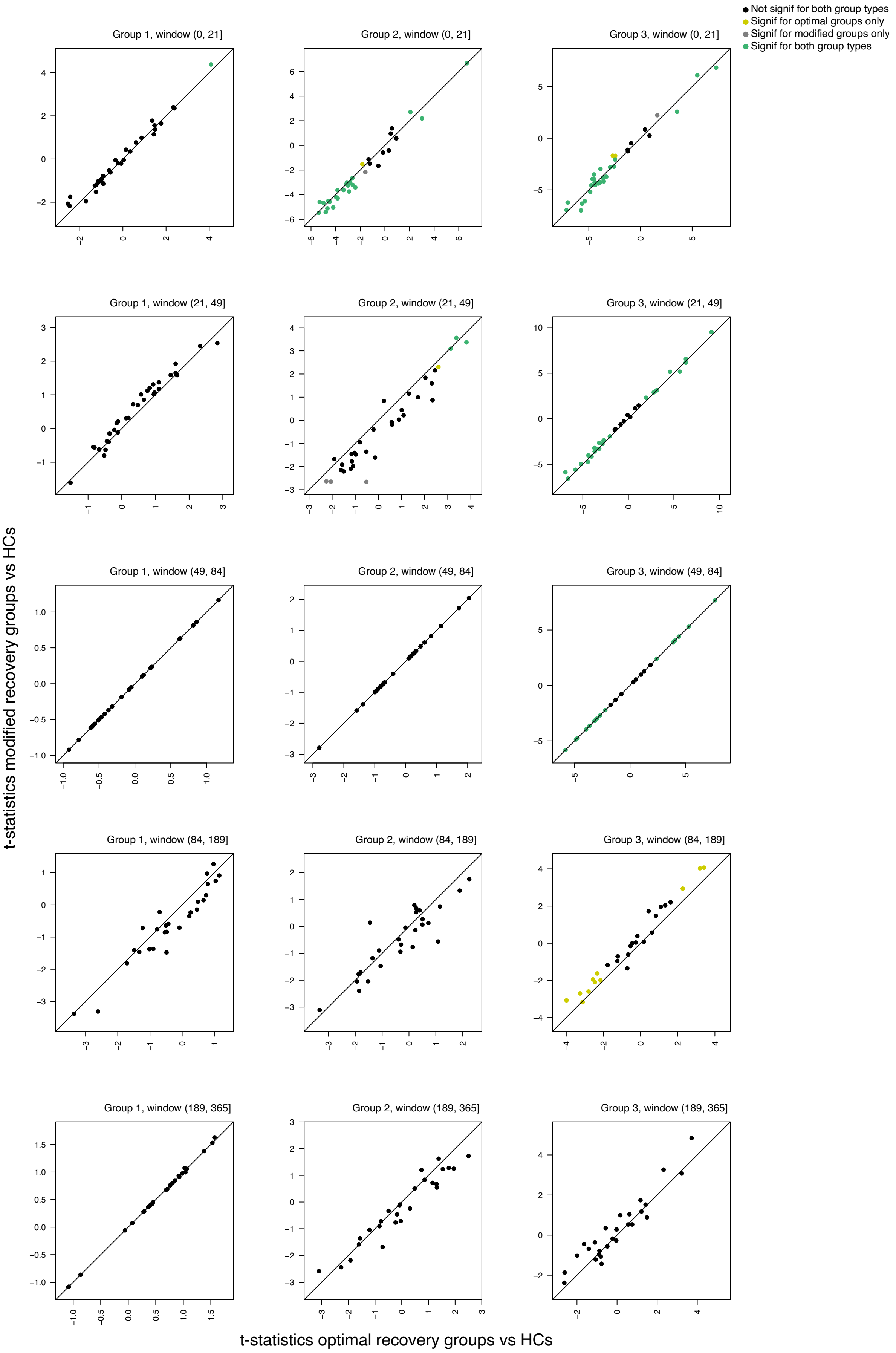

Supplementary Figure 7: **Sensitivity of long-term cell-subset differential abundance estimates to misassignment of CovPs to recovery groups.** Scatterplots comparing the estimates of Fig. 3 of the main text based on the optimal group assignment (x-axis) and the modified group assignment (y-axis) for the 5 time windows (rows) and the three recovery groups (columns). Colors indicate significance reached under such assignments at FDR 5%.

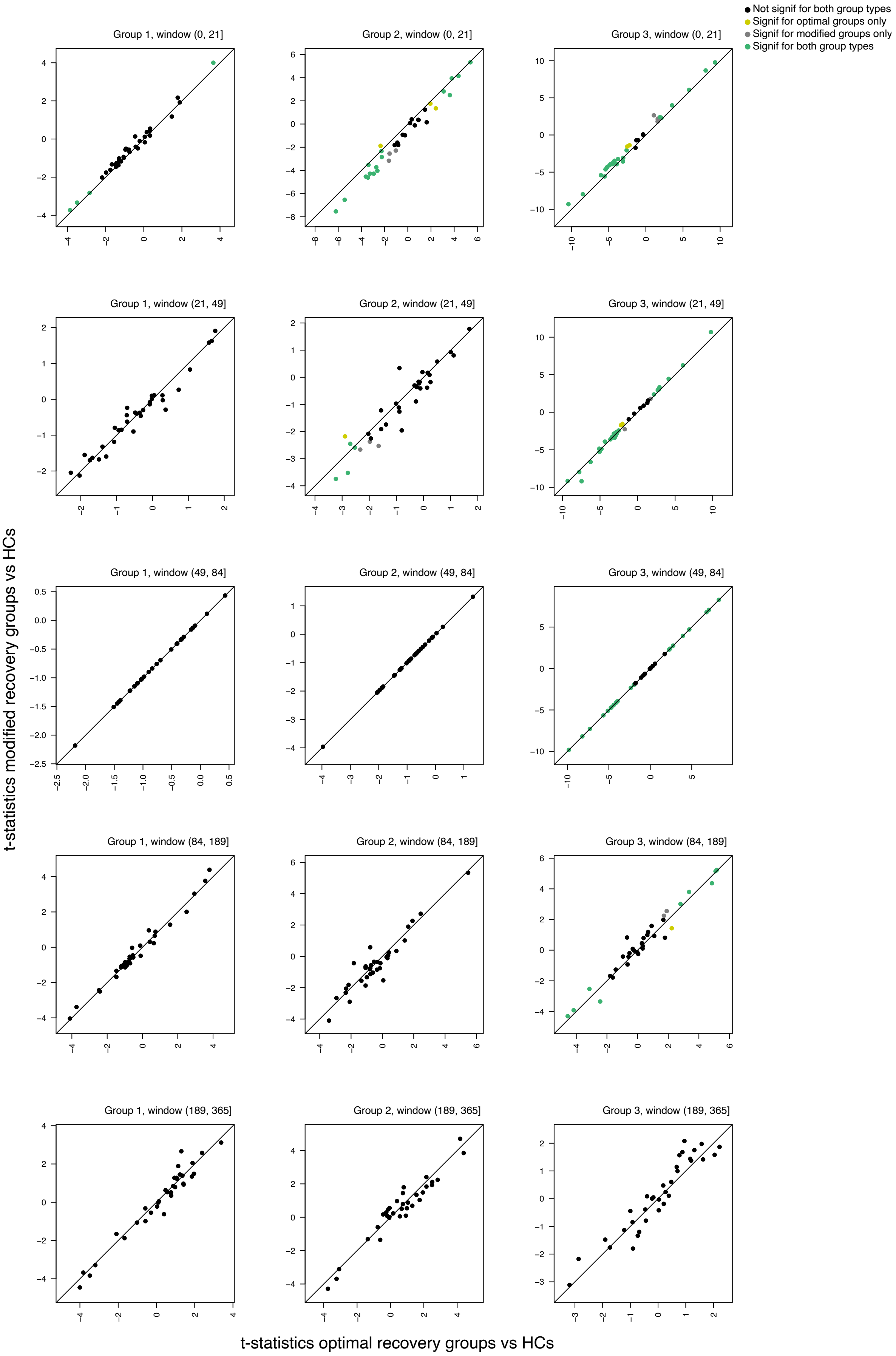

Supplementary Figure 8: **Sensitivity of long-term polar-metabolite differential abundance estimates to misassignment of CovPs to recovery groups.** See caption of Supplementary Fig. 7 for labeling details.

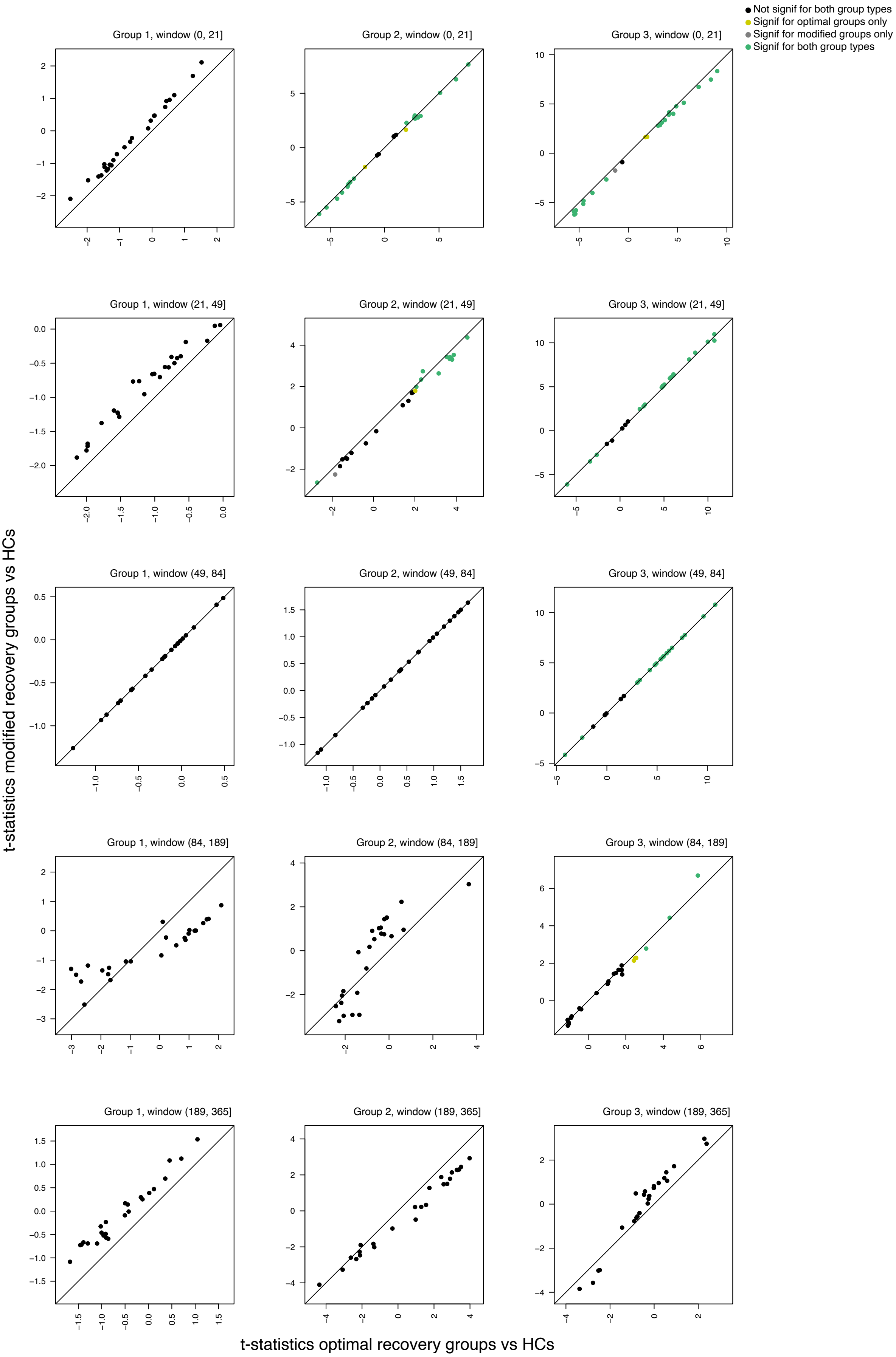

Supplementary Figure 9: **Sensitivity of long-term glyco- & lipoprotein differential abundance estimates to misassignment of CovPs to recovery groups.** See caption of Supplementary Fig. 7 for labeling details.

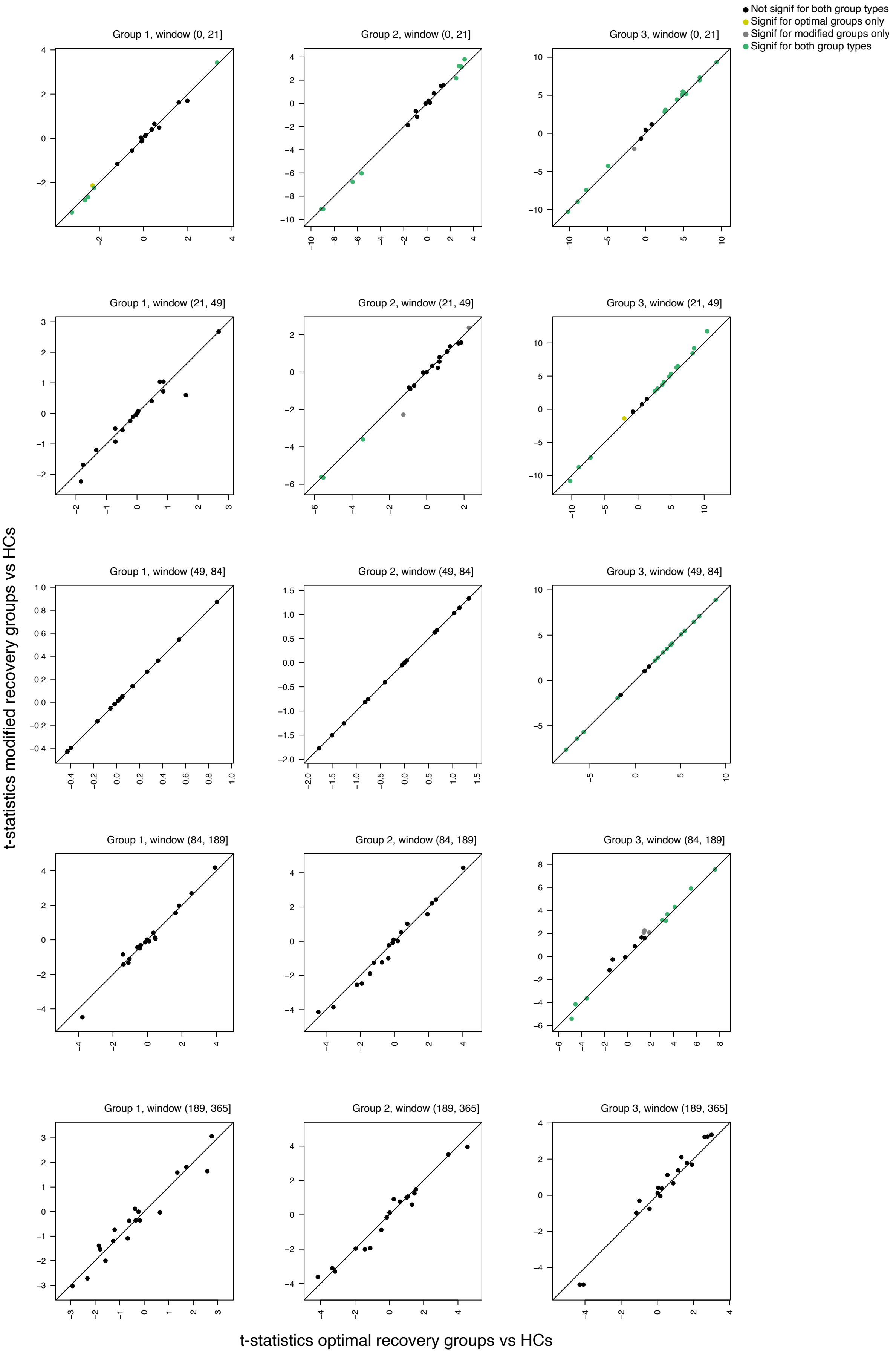

Supplementary Figure 10: **Sensitivity of long-term metabolic-ratio differential abundance estimates to misassignment of CovPs to recovery groups.** See caption of Supplementary Fig. 7 for labeling details.

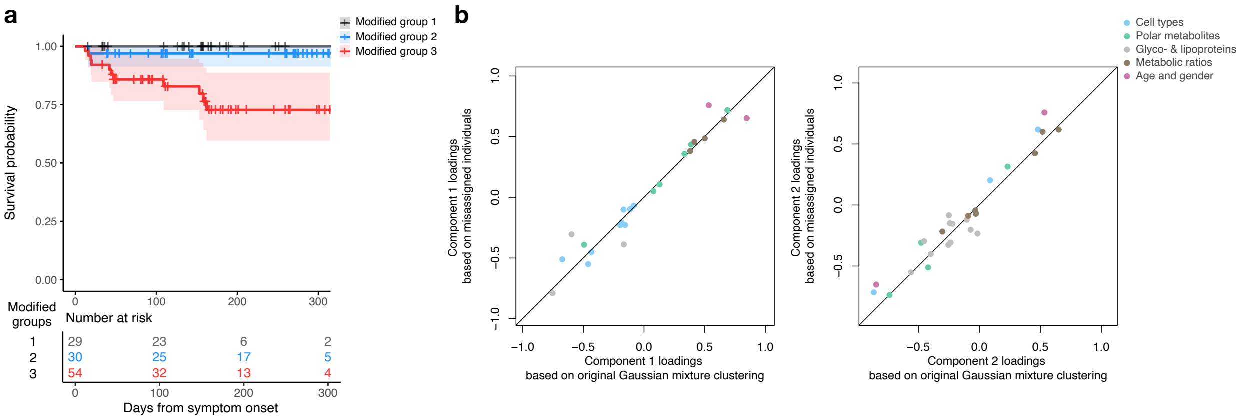

Supplementary Figure 11: **Sensitivity to CovPs misassignment for survival and prediction analyses.** **a.** Kaplan–Meier survival curves based on the modified recovery-group assignment, with 95% confidence bands. **b.** Scatterplots comparing the estimated loadings of Fig. 5 of the main text based on the optimal group assignment (x-axis) with those based on the modified group assignment (y-axis) for the two signatures (latent components 1 and 2, left and right, respectively).

Supplementary Data 4. Complement on the long-term symptom analyses

The self-reported symptom data collected from CovPs between 2 and 11 months post disease onset consisted in ordinal variables on the Likert scale, ranging from 0 (no symptomatology) to 5 (extreme symptomatology). The definition of the scores for each symptom is provided in the Source Data Table 1.

Supplementary Fig. 12 shows the correlation and partial correlation among scores. Neurological symptoms tend to group together and be associated (upper part dendrogram in Supplementary Fig. 13 & top left block significantly correlated after multiplicity adjustment in Supplementary Fig. 12a). Supplementary Fig. 13 shows the scores collected for the CovPs, by recovery groups. The data are sparse and entail substantial patient-to-patient variability. The scree plot was used to determine the number of factors in the principal axis factor analysis (Supplementary Fig. 14a). A factor graph was then reconstructed from the first two retained factors (14b). The neurological nature of the first latent factor can be seen from the graph (which provides an alternative visualization to that of Fig. 4a of the main text).

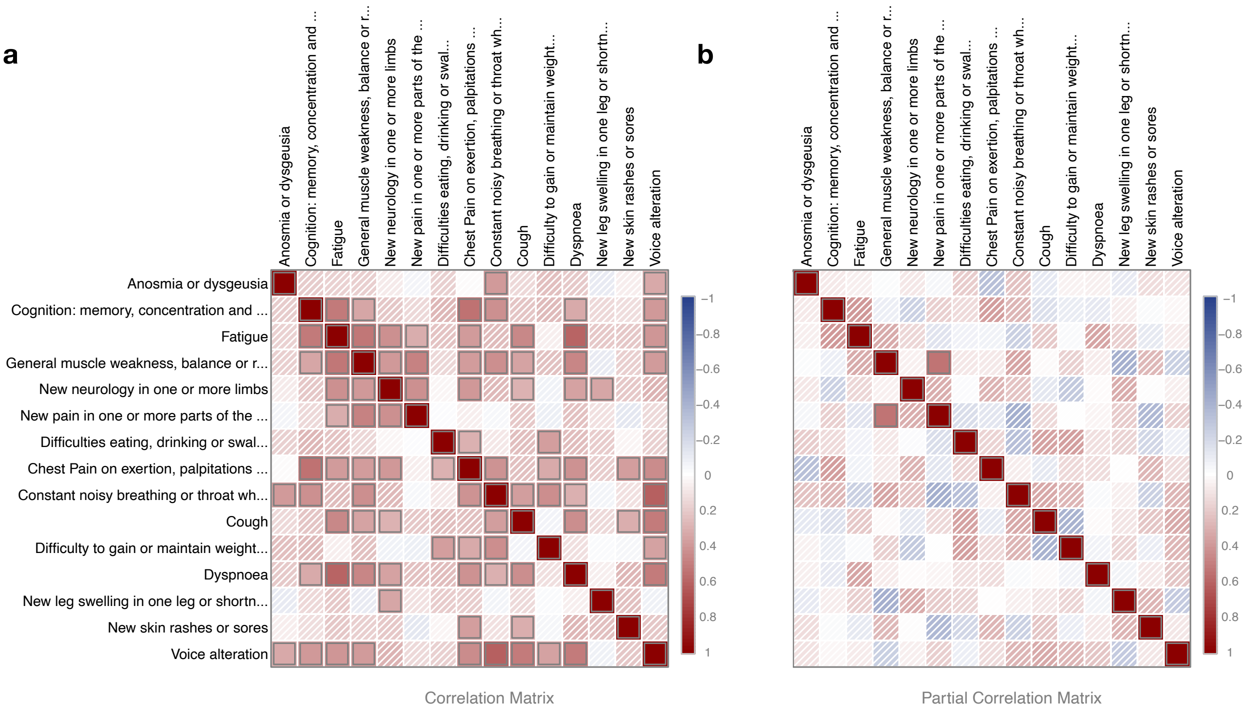

Supplementary Figure 12: **Correlation structure of long-term symptoms. a.** Spearman rank correlation. Solid colors indicate significance at FDR 5%. **b.** Partial correlation. Solid colors indicate significance at FDR 5%.

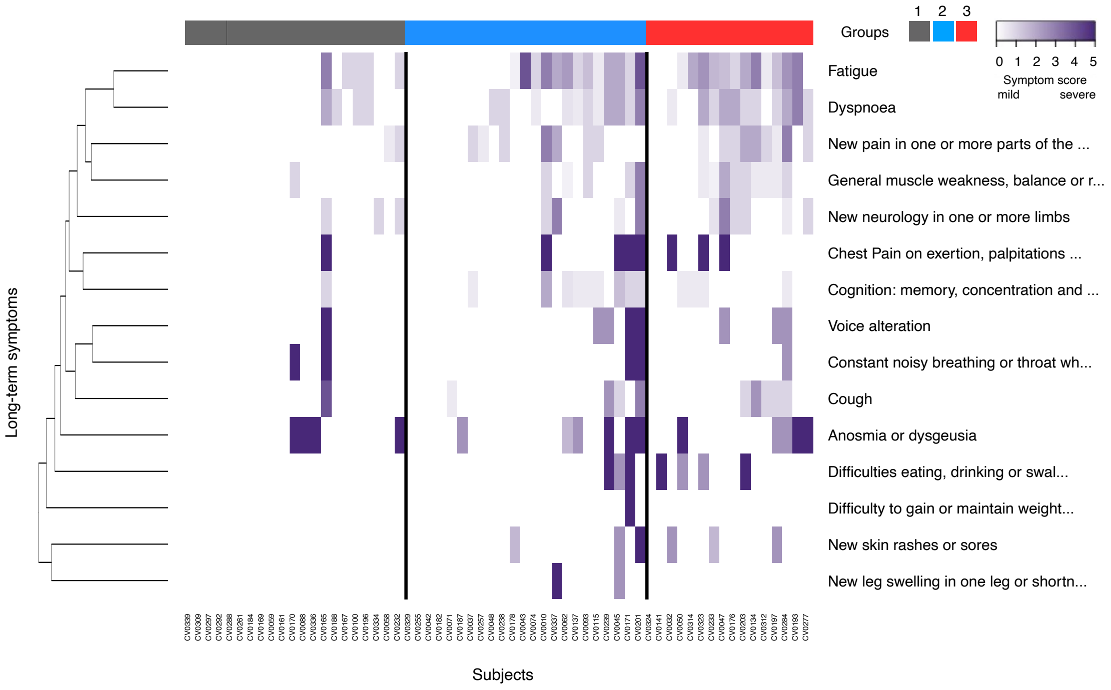

Supplementary Figure 13: **Long-term symptom data by recovery groups.** Analyzed symptoms (rows), excluding persisting fever which was not experienced by any CovP, against CovPs analyzed in the FPCA framework for which at least one questionnaire was collected (columns).

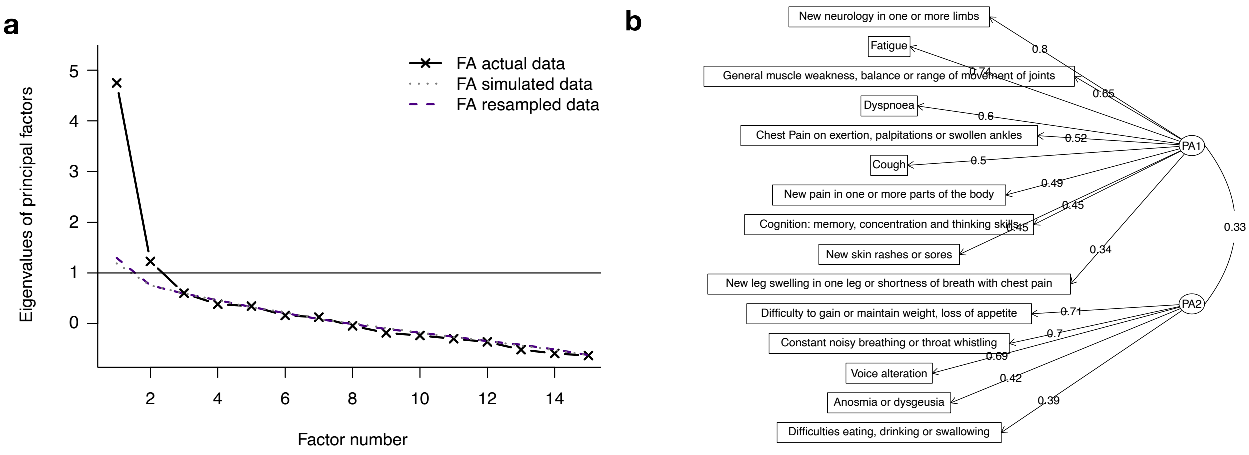

Supplementary Figure 14: **Principal axis latent factor analysis of long-term symptoms**. **a.** Eigenvalue scree plot, with comparison of eigenvalues of observed data with those of a random data matrix (simulated or resampled) of the same size as the original matrix. Sharp breaks suggest the appropriate number of factors to extract. **b.** Factor loading graph for the major loadings on each of the two factors (PA1 & PA2). The edge connecting the two factors indicates their correlation.
