# Supplementary material for: A patient-centric modeling framework captures recovery from SARS-CoV-2 infection
Source: Nat Immunol. 2023 Jan 30;24(2):349–58. doi: 10.1038/s41590-022-01380-2 (PMC9892000; doi:10.1038/s41590-022-01380-2)
Supplement: Supplementary file 2 — Reporting Summary [file 41590_2022_1380_MOESM2_ESM.pdf]

nature portfolio

Corresponding author(s): Hélène Ruffieux and Christoph Hess

Last updated by author(s): Oct 30, 2022

Reporting Summary

Nature Portfolio wishes to improve the reproducibility of the work that we publish. This form provides structure for consistency and transparency in reporting. For further information on Nature Portfolio policies, see our Editorial Policies and the Editorial Policy Checklist.

Statistics

For all statistical analyses, confirm that the following items are present in the figure legend, table legend, main text, or Methods section.

n/a	Confirmed	
☐	☒	The exact sample size (*n*) for each experimental group/condition, given as a discrete number and unit of measurement
☐	☒	A statement on whether measurements were taken from distinct samples or whether the same sample was measured repeatedly
☐	☒	The statistical test(s) used AND whether they are one- or two-sided<br>*Only common tests should be described solely by name; describe more complex techniques in the Methods section.*
☐	☒	A description of all covariates tested
☐	☒	A description of any assumptions or corrections, such as tests of normality and adjustment for multiple comparisons
☐	☒	A full description of the statistical parameters including central tendency (e.g. means) or other basic estimates (e.g. regression coefficient) AND variation (e.g. standard deviation) or associated estimates of uncertainty (e.g. confidence intervals)
☐	☒	For null hypothesis testing, the test statistic (e.g. *F*, *t*, *r*) with confidence intervals, effect sizes, degrees of freedom and *P* value noted<br>*Give P values as exact values whenever suitable.*
☒	☐	For Bayesian analysis, information on the choice of priors and Markov chain Monte Carlo settings
☒	☐	For hierarchical and complex designs, identification of the appropriate level for tests and full reporting of outcomes
☐	☒	Estimates of effect sizes (e.g. Cohen's *d*, Pearson's *r*), indicating how they were calculated

Our web collection on statistics for biologists contains articles on many of the points above.

Software and code

Policy information about availability of computer code

Data collection	FlowJo v10.2, Target Analysis for Screening Quantification (TASQ) software v2.2, MassLynx v4.2, statistical software R v4.1.0
Data analysis	The statistical software R v4.1.0 was used for all statistical analyses, as stated in the Methods section

For manuscripts utilizing custom algorithms or software that are central to the research but not yet described in published literature, software must be made available to editors and reviewers. We strongly encourage code deposition in a community repository (e.g. GitHub). See the Nature Portfolio guidelines for submitting code & software for further information.

Data

Policy information about availability of data

All manuscripts must include a data availability statement. This statement should provide the following information, where applicable:

- Accession codes, unique identifiers, or web links for publicly available datasets
- A description of any restrictions on data availability
- For clinical datasets or third party data, please ensure that the statement adheres to our policy

The data and metadata for the early (0-3 months) follow-up are available at NIHR CITIID COVID-19 Cohort (https://www.covid19cellatlas.org/patient/citiid/).

Human research participants

Policy information about studies involving human research participants and Sex and Gender in Research.

Reporting on sex and gender	SARS-CoV-2 positive participants were recruited almost equally from both genders (M:53%, F:47%), as were healthy controls (M:56%, F:44%). Our findings apply to both genders. All study participants provided written informed consent prior to enrolment. Gender was included as a covariate where appropriate in statistical analyses.
Population characteristics	Age – SARS-CoV-2 positive participants: mean 52.34 (sd 17.93), healthy controls: mean 42.16 (sd 15.16). For gender please see above. Ethnicity: 66.4% white, 5.3% asian, 7%. other, 21% NA. Other infections: 11.5% proven, 23.9% suspected, 64.6% not suspected. Hospital outcome: 63.0% discharged, 22.2% hospital or other facility, 14.8% death. Comprehensive clinical metadata/information about participants is available at https://www.covid19cellatlas.org/patient/citiid/
Recruitment	Study participants were patients attending Addenbrooke's Hospital, Royal Papworth Hospital NHS Foundation Trust or Cambridge and Peterborough Foundation Trust with a confirmed diagnosis of SARS-CoV-2 infection, as well as SARS-CoV-2 positive health care workers recruited from a staff screening program. Recruitment process was designed to avoid potential sources of selection bias, in particular, patients were distributed across a range of ages, disease severities and both genders (see manuscript).
Ethics oversight	Ethics approval was obtained from the East of England – Cambridge Central Research Ethics Committee ("NIHR BioResource" REC ref 17/EE/0025, and "Genetic variation AND Altered Leucocyte Function in health and disease - GANDALF" REC ref 08/H0308/176).

Note that full information on the approval of the study protocol must also be provided in the manuscript.

Field-specific reporting

Please select the one below that is the best fit for your research. If you are not sure, read the appropriate sections before making your selection.

☒ Life sciences ☐ Behavioural & social sciences ☐ Ecological, evolutionary & environmental sciences

For a reference copy of the document with all sections, see nature.com/documents/nr-reporting-summary-flat.pdf

Life sciences study design

All studies must disclose on these points even when the disclosure is negative.

Sample size	Patients were recruited so as to cover a broad range of clinical severities and multiple samples per patient were collected over a year post symptom onset. Specifically, the cohort involved: n = 18 asymptomatic healthy workers (class A); n = 40 symptomatic healthy workers (still working or self-isolating, class B); n = 50 patients who presented to hospital but never required oxygen supplementation (class C); n = 38 patients who were admitted to hospital and whose maximal respiratory support was supplemental oxygen (class D); n = 69 patients who at some point required assisted ventilation (class E), n=45 healthy controls (SARS-CoV-2 PCR-negative hospital staff members with a negative serology). The sample sizes were sufficient to reach statistical significance in our analyses, after appropriate multiplicity correction.
Data exclusions	Six patients were excluded because of extreme confounding comorbidities, as described in doi: 10.1016/j.immuni.2021.05.010. A standard boxplot rule was applied to discard extreme samples, i.e., with >20% of their measurements falling outside the Tukey outer fences. Following this procedure, 2 immune cell type samples (1.1%) and 10 metabolomic samples (1.6%) were removed from all downstream analyses (all glyco- and lipoprotein samples were retained).
Replication	Standard population-level findings obtained from other cohorts were confirmed in ours. The performance of our prediction framework was confirmed in an independent sample of our cohort.
Randomization	Not applicable as this was not an intervention study.
Blinding	Not applicable as this was not an intervention study.

Reporting for specific materials, systems and methods

We require information from authors about some types of materials, experimental systems and methods used in many studies. Here, indicate whether each material, system or method listed is relevant to your study. If you are not sure if a list item applies to your research, read the appropriate section before selecting a response.

Materials & experimental systems

n/a	Involved in the study	
☐	☒	Antibodies
☒	☐	Eukaryotic cell lines
☒	☐	Palaeontology and archaeology
☒	☐	Animals and other organisms
☐	☒	Clinical data
☒	☐	Dual use research of concern

Methods

n/a	Involved in the study	
☒	☐	ChIP-seq
☐	☒	Flow cytometry
☒	☐	MRI-based neuroimaging

Antibodies

Antibodies used	Data collected at additional timepoints, following the same procedure as in doi: 10.1016/j.immuni.2021.05.010
Validation	Data collected at additional timepoints, following the same procedure in doi: 10.1016/j.immuni.2021.05.010

Clinical data

Policy information about clinical studies
All manuscripts should comply with the ICMJE guidelines for publication of clinical research and a completed CONSORT checklist must be included with all submissions.

Clinical trial registration	No clinical trial was performed - prospective observational cohort study
Study protocol	n/a
Data collection	n/a
Outcomes	n/a

Flow Cytometry

Plots

Confirm that:

☒ The axis labels state the marker and fluorochrome used (e.g. CD4-FITC).

☒ The axis scales are clearly visible. Include numbers along axes only for bottom left plot of group (a 'group' is an analysis of identical markers).

☒ All plots are contour plots with outliers or pseudocolor plots.

☒ A numerical value for number of cells or percentage (with statistics) is provided.

Methodology

Sample preparation	Data collected at additional timepoints, following the same procedure as in doi: 10.1016/j.immuni.2021.05.010
Instrument	Data collected at additional timepoints, following the same procedure as in doi: 10.1016/j.immuni.2021.05.010
Software	Data collected at additional timepoints, following the same procedure as in doi: 10.1016/j.immuni.2021.05.010
Cell population abundance	Data collected at additional timepoints, following the same procedure as in doi: 10.1016/j.immuni.2021.05.010
Gating strategy	Data collected at additional timepoints, following the same procedure as in doi: 10.1016/j.immuni.2021.05.010

☐ Tick this box to confirm that a figure exemplifying the gating strategy is provided in the Supplementary Information.
