# Supplementary material for: A patient-centric modeling framework captures recovery from SARS-CoV-2 infection
Source: Nat Immunol. 2023 Jan 30;24(2):349–58. doi: 10.1038/s41590-022-01380-2 (PMC9892000; doi:10.1038/s41590-022-01380-2)
Supplement: Supplementary file 3 — Cohort characteristics by recovery groups 1–3. ‘Length of hospital stay’ is the number of days from hospital admission to discharge, transfer or death in hospital. sd, standard deviation. [file 41590_2022_1380_MOESM3_ESM.pdf]

Recovery group	1	2	3
**Number of subjects:**	29	30	54
**Gender:**			
male %	24.14	60.00	72.22
**Age:**			
years, mean (sd)	37.17 (14.02)	56.60 (13.16)	57.72 (15.10)
**BMI:**			
kg/m^2, mean (sd)	27.22 (3.59)	29.21 (4.26)	29.13 (6.76)
NA %	93.10	23.33	16.67
**Ethnicity:**			
white %	72.41	83.33	53.70
asian %	6.90	3.33	5.56
other %	6.90	3.33	9.26
NA %	13.79	10.00	31.48
**Severity class:**			
B, screening symptomatic %	86.21	13.33	5.56
C, hospital no O2 required %	13.79	30.00	9.26
D, hospital supplemental O2 %	0.00	40.00	9.26
E, hospital assisted ventilation %	0.00	16.67	75.93
**Secondary infection:**			
proven %	0.00	13.33	16.67
suspected %	0.00	36.67	29.63
**Days from COVID-19 symptoms to enrollment:**			
years, mean (sd)	6.55 (3.05)	10.67 (7.15)	19.93 (12.69)
**Admitted to ITU:**			
%	0.00	23.33	70.37
**Hospital outcome (if class C, D or E):**			
discharged %	75.00	92.31	47.06
hospital or other facility %	25.00	7.69	29.41
deceased in hospital %	0.00	0.00	23.53
**Length of hospital stay (if class C, D or E):**			
days, median	6.50	7.50	25.00
**Full physical and mental recovery:**			
yes % of subjects asked (self-reported)	70.00	28.57	33.33
NA % total	31.03	30.00	72.22
