# Supplementary material for: A patient-centric modeling framework captures recovery from SARS-CoV-2 infection
Source: Nat Immunol. 2023 Jan 30;24(2):349–58. doi: 10.1038/s41590-022-01380-2 (PMC9892000; doi:10.1038/s41590-022-01380-2)
Supplement: Supplementary file 5 — Supplementary Note [file 41590_2022_1380_MOESM5_ESM.pdf]

The CITIID-NIHR BioResource COVID-19 Collaboration Authorship Banner

Stephen Baker[2,6], John R. Bradley[1,3,6,10,14], Patrick Chinnery[3,22,23], Daniel Cooper[10, 24], Gordon Dougan[2,6], Ian Goodfellow[7], Ravindra Gupta[2,6,12,15], Nathalie Kingston[3,4], Paul J. Lehner[2,6,12], Paul A. Lyons[2,6], Nicholas J. Matheson[2,6,12,26], Caroline Saunders[9], Kenneth G. C. Smith[2,6], Charlotte Summers[6,11,25], James Thaventhiran[18], M. Estee Torok [6,12,13], Mark R. Toshner[6,8,25], Michael P. Weekes[2,6,12,27], Gisele Alvio[9], Sharon Baker[9], Areti Bermperi[9], Karen Brookes[9], Ashlea Bucke[9,10], Jo Calder[9,10], Laura Canna[9,10], Cherry Sanchez[9,10], Isabel Cruz[9], Ranalie de Jesus[9], Katie Dempsey[9], Giovanni Di Stephano[9], Jason Domingo[9], Anne Elmer[9], Julie Harris[9,10,29], Sarah Hewitt[9,10], Heather Jones[9], Sherly Jose[9], Jane Kennet[9,27], Yvonne King[10], , Jenny Kourampa[9], Emily Li[9,10], Caroline McMahon[9], Anne Meadows[9,10], Vivien Mendoza[9], Criona O'Brien[9,10,29], Charmain Ocaya[9], Ciro Pasquale[9], Marlyn Perales[9], Jane Price[9,10], Rebecca Rastall[9,10], Carla Ribeiro[9], Jane Rowlands[9,10], Valentina Ruffolo[9,10], Hugo Tordesillas[9,10], Phoebe Vargas[9], Bensi Vergese[9], Laura Watson[9], Jieniean Worsley[9], Julie-Ann Zerrudo[9], Laura Bergamashi[2,6], Ariana Betancourt[2,6], Georgie Bower, Ben Bullman, Chiara Cossetti[27], Aloka de Sa[2,6], Benjamin J. Dunmore[6], Maddie Epping[2,6], Stuart Fawke[19], Stefan Gräf[3,6], Richard Grenfell, Andrew Hinch[6], Josh Hodgson[6], Christopher Huang[19], Oisin Huhn[30], Kelvin Hunter[2,6], Isobel Jarvis[6], Emma Jones[19], Maša Josipović[31], Ekaterina Legchenko[6], Daniel Lewis[6], Joe Marsden[19], Jennifer Martin[19], Federica Mescia[2,6], Francesca Nice[19], Ciara O'Donnell[19], Ommar Omarjee[2], Marianne Perera[19], Linda Pointon[19], Nicole Pond[6], Nathan Richoz[6], Nika Romashova[19], Natalia Savinykh[6], Rahul Sharma[19], Joy Shih, Mateusz Strezlecki[19], Rachel Sutcliffe[6], Tobias Tilly[6], Zhen Tong[6], Carmen Treacy[6], Lorinda Turner[2,6], Jennifer Wood[6], Marta Wylot[19], John Allison[3,4], Heather Biggs[3,17], Helen Butcher[3,5], Daniela Caputo[3,5], Matt Chandler[3,5], Patrick Chinnery[3,22,23], Debbie Clapham-Riley[3,5], Eleanor Dewhurst[3,5], Christian Fernandez[3,] Anita Furlong[3,5], Barbara Graves[3,5], Jennifer Gray[3,5], Sabine Hein[3,5], Tasmin Ivers[3,5], Emma Le Gresley[3,5], Rachel Linger[3,5], Mary Kasanicki[3,10], Rebecca King[3,5], Sarah Meloy[3,5], Alexei Moulton[3,5], Francesca Muldoon[3,5], Nigel Ovington[3,4], Sofia Papadia[3,5], Christopher J. Penkett[3,4], Isabel Phelan[3,5], Venkatesh Ranganath[3,4], Roxana Paraschiv[3,4], Abigail Sage[3,5], Jennifer Sambrook[3,4], Ingrid Scholtes[3,5], Katherine Schon[3,16,17], Hannah Stark[3,5], Kathleen E. Stirrups[3,4], Paul Townsend[3,4], Neil Walker[3,4], Jennifer Webster[3,5], Mayurun Selvan[28], Petra, Polgarova[11],Sarah L. Caddy[2,6], Laura G. Caller[19,20], Yasmin Chaudhry[7], Martin D. Curran[21], Theresa Feltwell[6], Stewart Fuller[19], Iliana Georgana[7], Grant Hall[7], William L. Hamilton[6,12,13], Myra Hosmillo[7], Charlotte J. Houldcroft[6], Rhys Izuagbe[7], Aminu S. Jahun[7], Fahad A. Khokhar[2,6], Anna G. Kovalenko[7],

Luke W. Meredith[7], Surendra Parmar[21], Malte L. Pinckert[7], Anna Yakovleva[7], Emily C. Horner[18], Lucy Booth[18], Alexander Ferreira[18], Rebecca Boston[18], Robert Hughes[18], Juan Carlos Yam Puc[18], Nonantzin Beristain-Covarrubias[18], Maria Rust[18], Thevinya Gurugama[18], Lihinya Gurugama[18], Thomas Mulroney[18], Sarah Spencer[18], Zhaleh Hosseini[18], Kate Williamson[18].

[1]NIHR Cambridge Biomedical Research Centre, Cambridge Biomedical Campus, Cambridge, UK

[2]Cambridge Institute of Therapeutic Immunology and Infectious Disease (CITIID), Jeffrey Cheah Biomedical Centre, Cambridge Biomedical Campus, Cambridge, UK

[3]NIHR BioResource, Cambridge University Hospitals NHS Foundation Trust, Cambridge Biomedical Campus, Cambridge, UK

[4]Department of Haematology, School of Clinical Medicine, University of Cambridge, Cambridge Biomedical Campus, Cambridge, UK

[5]Department of Public Health and Primary Care, School of Clinical Medicine, University of Cambridge, Cambridge Biomedical Campus, Cambridge, UK

[6]Department of Medicine, School of Clinical Medicine, University of Cambridge, Cambridge Biomedical Campus, Cambridge, UK

[7]Division of Virology, Department of Pathology, University of Cambridge, Cambridge, UK

[8]Royal Papworth Hospital NHS Foundation Trust, Cambridge, UK

[9]NIHR Cambridge Clinical Research Facility (CRF), and CRF Outreach Team, Addenbrookes Hospital, Cambridge University Hospitals NHS Foundation Trust, Cambridge, UK

[10]Addenbrooke's Hospital, Cambridge University Hospitals NHS Foundation Trust, Cambridge Biomedical Campus, Cambridge, UK

[11]Intensive Care Unit, Addenbrooke's Hospital, Cambridge University Hospitals NHS Foundation Trust, Cambridge Biomedical Campus, Cambridge, UK

[12]Department of Infectious Diseases, Addenbrooke's Hospital, Cambridge University NHS Hospitals Foundation Trust, Cambridge, UK

[13]Department of Microbiology, Addenbrooke's Hospital, Cambridge University NHS Hospitals Foundation Trust, Cambridge, UK

[14]Department of Renal Medicine, Addenbrooke's Hospital, Cambridge University Hospitals NHS Foundation Trust, Cambridge, UK
[15]Africa Health Research Institute, Durban, South Africa
[16]Clinical Genetics, Addenbrooke's Hospital, Cambridge University Hospitals NHS Foundation Trust, Cambridge, UK
[17]Department of Clinical Neurosciences, School of Clinical Medicine, University of Cambridge, Cambridge Biomedical Campus, Cambridge, UK
[18]MRC Toxicology Unit, Gleeson Building, Tennis Court Road, Cambridge, UK
[19]University of Cambridge, Cambridge, UK
[20]The Francis Crick Institute, London, UK
[21]Public Health England, Clinical Microbiology and Public Health Laboratory, Cambridge, UK
[22]Department of Clinical Neurosciences, School of Clinical Medicine, University of Cambridge, Cambridge Biomedical Campus, Cambridge, UK
[23]Medical Research Council Mitochondrial Biology Unit, Cambridge Biomedical Campus, Cambridge, UK
[24]Global and Tropical Health Division, Menzies School of Heath Research and Charles Darwin University, Darwin, Northern Territory, Australia
[25]Heart and Lung Research Institute, Cambridge Biomedical Campus, Cambridge, UK
[26]NHS Blood and Transplant, Cambridge, UK
[27]Cambridge Institute for Medical Research, Biomedical Campus, Hills Rd, Cambridge UK
[28] Department of Respiratory Medicine, Cambridge University Hospitals NHS Foundation Trust, Cambridge, UK
[29] Department of Paediatric Medicine, Cambridge University Hospitals NHS Foundation Trust, Cambridge, UK
[30]Department of Paediatric Medicine, Cambridge University Hospitals NHS Foundation Trust, Cambridge, UK
[31]Metabolic Research Laboratories, Wellcome Trust-Medical Research Council Institute of Metabolic Science, University of Cambridge, Cambridge CB2 0QQ, UK
